# Endogenously produced catecholamines improve the regulatory function of TLR9-activated B cells

**DOI:** 10.1371/journal.pbio.3001513

**Published:** 2022-01-24

**Authors:** Nadine Honke, Torsten Lowin, Birgit Opgenoorth, Namir Shaabani, Alexander Lautwein, John R. Teijaro, Matthias Schneider, Georg Pongratz

**Affiliations:** 1 Department of Rheumatology, Hiller Research Center Rheumatology, University Hospital Düsseldorf, Germany; 2 Department of Immunology and Microbial Science, The Scripps Research Institute, La Jolla, California, United States of America; Tokyo Medical and Dental University Medical Research Institute, JAPAN

## Abstract

The sympathetic nervous system (SNS) contributes to immune balance by promoting anti-inflammatory B cells. However, whether B cells possess a self-regulating mechanism by which they modulate regulatory B cell (Breg) function is not well understood. In this study, we investigated the ability of B cells to synthesize their own catecholamines upon stimulation with different B cell activators and found that expression of the enzyme tyrosine hydroxylase (TH), required to generate catecholamines, is up-regulated by Toll-like receptor (TLR)9. This TLR9-dependent expression of TH correlated with up-regulation of adrenergic receptors (ADRs), enhanced interleukin (IL)-10 production, and overexpression of the co-inhibitory ligands programmed death ligand 1 (PD-L1) and Fas ligand (FasL). Moreover, concomitant stimulation of ß1-3-ADRs together with a B cell receptor (BCR)/TLR9 stimulus clearly enhances the anti-inflammatory potential of Bregs to suppress CD4 T cells, a crucial population in the pathogenesis of autoimmune diseases, like rheumatoid arthritis (RA). Furthermore, TH up-regulation was also demonstrated in B cells during the course of collagen-induced arthritis (CIA), a mouse model for the investigation of RA. In conclusion, our data show that B cells possess an autonomous mechanism to modulate their regulatory function in an autocrine and/or paracrine manner. These findings help to better understand the function of B cells in the regulation of autoimmune diseases and the interplay of SNS.

## Abstract 2

The sympathetic nervous system produces neurotransmitters such as catecholamines which contribute to immune balance by promoting anti-inflammatory B cells. This study shows that mouse B cells can themselves synthesize, sense, and transport catecholamines, which in turn modulate regulatory B cell function in an autocrine and/or paracrine manner to suppress T cell proliferation.

## Introduction

The immune system requires checkpoints to prevent reactions against self-antigens. Faulty checkpoint inhibition can lead to multiple disorders and autoimmune diseases. While central tolerance is responsible for deleting autoreactive adaptive immune cells, peripheral tolerance is considered as a second-line checkpoint that controls lymphocytes that escaped deletion in primary lymphoid tissues. The sympathetic nervous system (SNS) might be one regulator of these processes since several studies showed that SNS influences the development and severity of autoimmune diseases, like rheumatoid arthritis (RA) [1]. Depleting the SNS in mice showed that the influence of the SNS is dependent on the stage of disease, with a pro-inflammatory role in the early phase and anti-inflammatory effect in the late phase of collagen-induced arthritis (CIA) [2]. Whereas the early pro-inflammatory SNS-mediated mechanisms are provided by unspecific systemic effects like increased provision of energy, higher blood and lymph flow, and an increased recruitment of lymphocytes, the late anti-inflammatory effects have been proposed to be more specific on adaptive immunity with involvement of regulatory cells and interleukin (IL)-10 [1,3,4]. In humans, an increased activity of the SNS is observed in patients with active RA [5] and electric stimulation of the vagus nerve, which results among other effects in the release of sympathetic neurotransmitters in the spleen, shows some alleviating effect [6].

A strong neuroimmune interaction has been shown in several previous studies [7–9]. For instance, sympathetic nerves that express tyrosine hydroxylase (TH) [10], the key enzyme in the biosynthesis of catecholamines, innervate lymphoid organs and gut-associated lymphoid tissues, where their nerve fiber endings are in close contact with immune cells like macrophages and T and B lymphocytes [11,12]. While immune cell–derived cytokines regulate the SNS, neurotransmitters such as norepinephrine (NE), epinephrine, and neuropeptides released from the SNS can affect the function of several immune cells [13,14]. Responses to catecholamines require the expression of G protein–coupled adrenergic receptors (ADRs). The impact of these receptors on immune cells has been previously characterized [15–17]. Among several ADR subtypes, the ß2-ADR was the most investigated with a bidirectional role in autoimmune diseases [18].

B cells are one of the major players in the pathogenesis of RA, since they can produce autoantibodies [19,20], and effector B cells activate CD4+ T helper cells, which may worsen the situation in the affected joints [21,22]. Regulatory B cells (Bregs) came into focus recently, since studies in mice have shown that they are not only essential to reduce chronic inflammation [23,24], but also effective in improving autoimmune disorders and maintaining tolerance [3,25–27]. Several different Breg subsets have been described in mice with similarities in their surface markers and function [28–34]. Besides IL-10 secretion, the immunosuppressive capacity or checkpoint activity of Bregs has also been associated with the production of IL-35 [35], tumor growth factor beta (TGF-ß) [36], and inhibitory costimulatory molecules, e.g., programmed death ligand 1 (PD-L1) [37,38] and Fas ligand (FasL) [39,40].

Since it is known that the function of Bregs is supported by SNS mediators, but, on the other hand, sympathetic nerve fibers are lost during chronic inflammation [10,41], we hypothesized that B cells have a self-sustained sympathetic backup mechanism to modulate their Breg function during chronic inflammation.

## Results

### B cells have their own machinery for catecholamine synthesis

**Fig 1:**
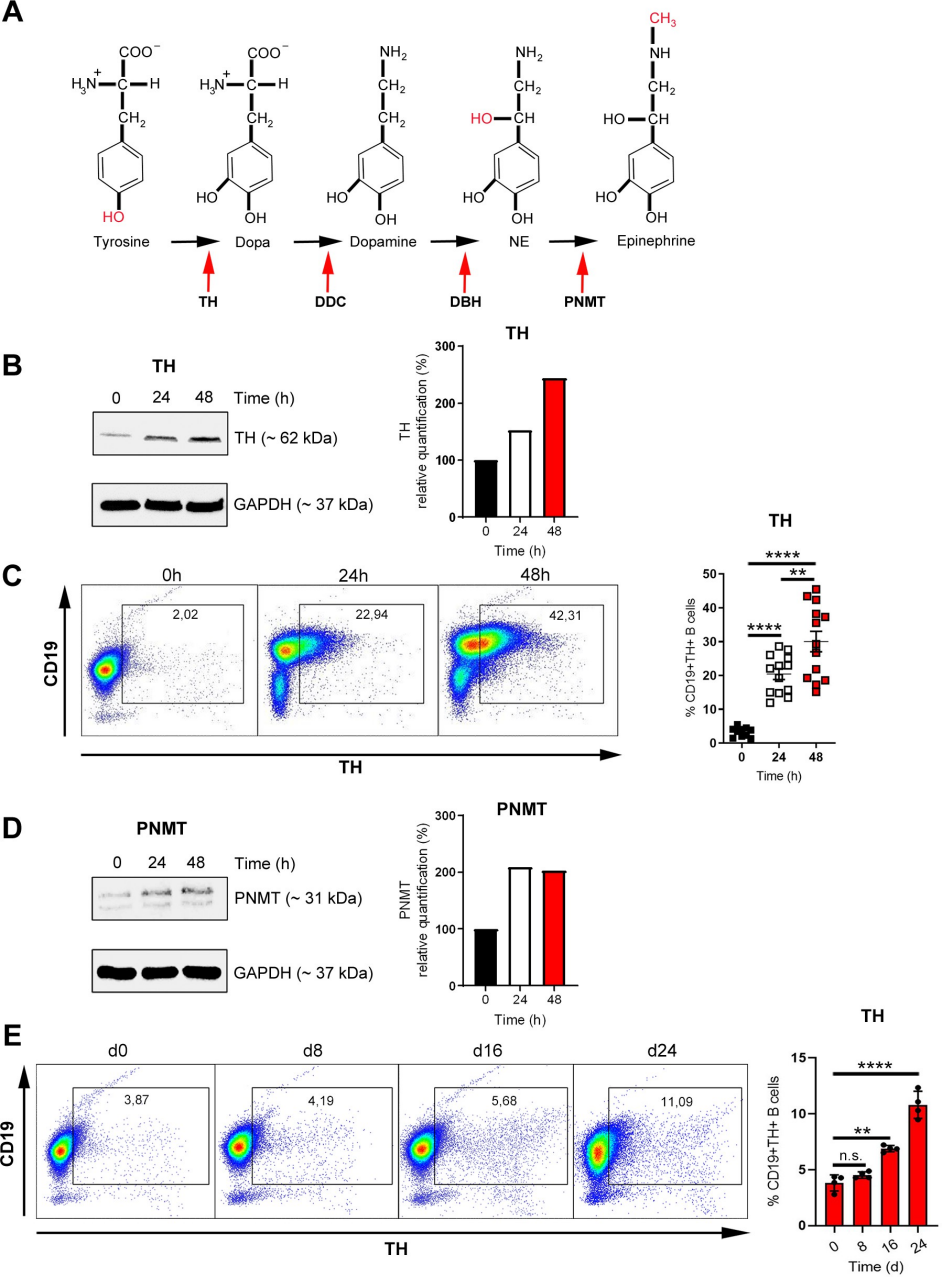
B cells have their own machinery for catecholamine synthesis. (**A**) Graphical depiction of the catecholamine biosynthetic pathway. (**B–D**) B cells were activated with anti-IgM/CpG for 24 or 48 h or left nonactivated. The expression of TH was analyzed by western blot (B) and flow cytometry (C; 0 h: *n* = 10; 24 h, 48 h: *n* = 13). Expression of PNMT was investigated by western blot (D). One representative blot of 3 is shown (B, D; *n* = 3). (**E**) DBA/1J mice were immunized with 100 μl of an emulsion of CII and CFA. After 0, 8, 16, and 24 d, splenic B cells from immunized mice were isolated by MACS and analyzed for TH expression by flow cytometry (*n* = 4). One representative dot plot (C, E) is shown. The TH and PNMT expression are indicated in relation to the housekeeping protein GAPDH (B, D). Full western blot images are available in S1 Fig. Data are pooled from 6 experiments (C). Statistical significance was determined by Brown–Forsythe and Welch 1-way ANOVA tests followed by Tamhane T2 multiple comparison test (C) and ordinary 1-way ANOVA followed by Dunnett multiple comparison test (E). n.s., not significant; *p < 0.5; **p < 0.01; ****p < 0.0001. For underlying data, see S1 Data. ANOVA, analysis of variance; CFA, complete Freund’s adjuvant; CII, type II collagen; GAPDH, glycerinaldehyd-3-phosphat-dehydrogenase; MACS, magnetic-activated cell sorting; PNMT, phenylethanolamine N-methyltransferase; TH, tyrosine hydroxylase.

Several enzymes are required to form catecholamines like norepinephrine (NE; noradrenaline) and/or epinephrine (adrenaline) starting from the amino acid tyrosine [42] (Fig 1A). The first and rate limiting enzyme in this process is TH, which converts tyrosine to L-3,4-dihydroxyphenylalanine (L-Dopa), whereas phenylethanolamine N-methyltransferase (PNMT), the terminal enzyme in this cascade, metabolizes NE to epinephrine (Fig 1A). First, we tested whether B cells express the necessary enzymes for biosynthesis of catecholamines. B cells were isolated from spleens of naive DBA/1J mice and activated T cell independently with anti-B cell receptor (BCR) IgM and the Toll-like receptor (TLR)9 agonist CpG. Not-activated B cells express low levels of TH, whereas activation augmented TH levels as assessed by western blot (Fig 1B, S1A Fig) and flow cytometry (Fig 1C). Similarly, activation of B cells with anti-IgM/CpG increased protein levels of PNMT (Fig 1D, S1B Fig), which converts NE to epinephrine. In order to assess whether an increase in B cell TH expression is specific for B cells from DBA/1J mice, splenic B cells from other mouse strains were additionally isolated and investigated for TH expression. B cells from C57BL/6 and BALB/c mice also increased TH expression after activation with anti-IgM/CpG in a similar manner as observed for DBA/1J mice (S2A Fig). Since catecholamines influence the severity of RA, we wondered whether B cells up-regulate TH during murine CIA, an autoimmune model for the investigation of an RA. CIA was induced in DBA/1J mice, and the expression of TH was monitored over time showing an increase during the course of arthritis (Fig 1E). This demonstrated that B cells activated in vitro or in vivo during CIA up-regulate enzymes, which are required to synthesize their own catecholamines independently of the SNS.

### Catecholamines are produced in a time-/stimulus-dependent way

**Fig 2:**
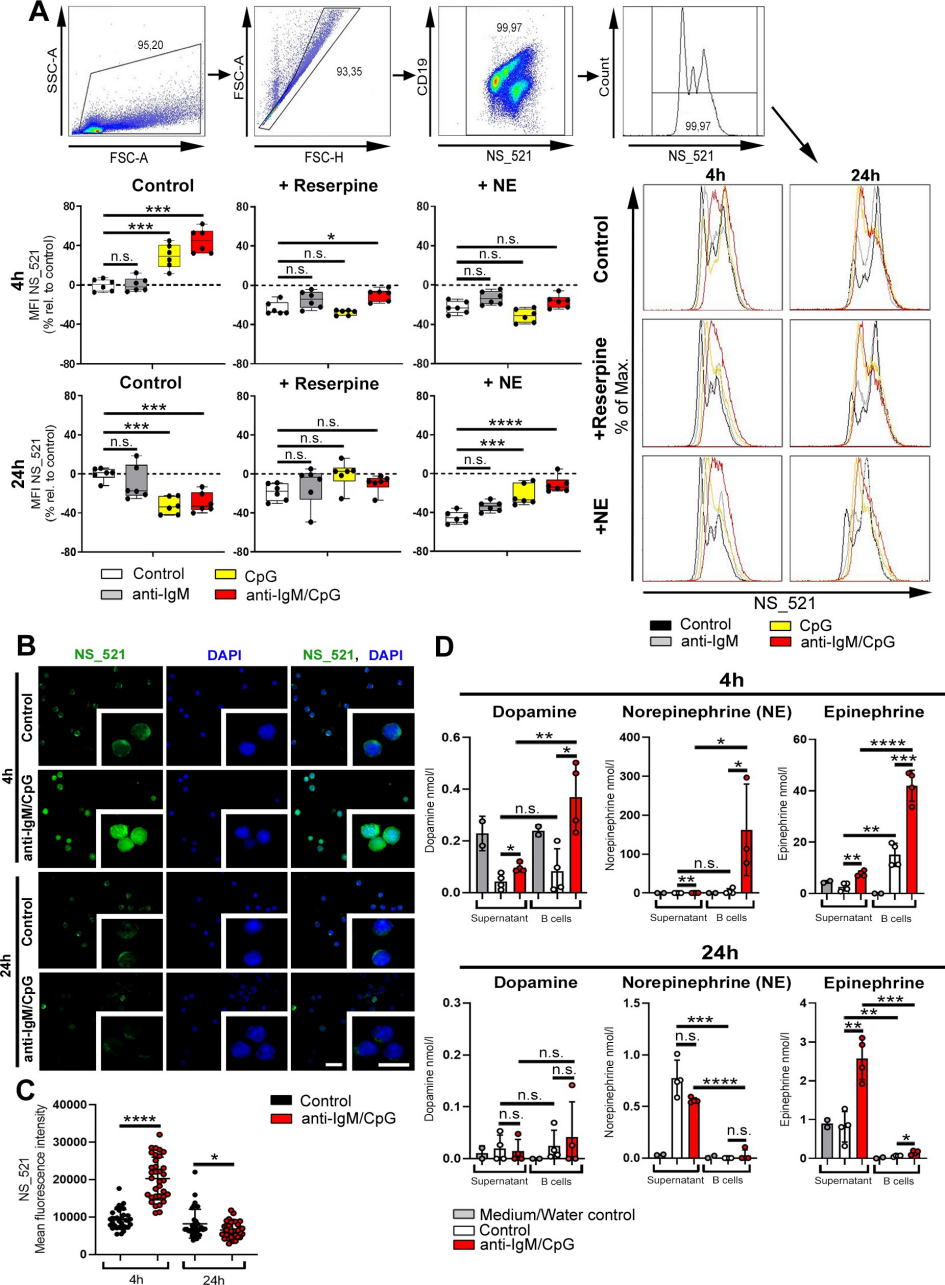
Catecholamines are produced in a time-/stimulus-dependent way. (**A**) B cells were activated for 4 h or 24 h with anti-IgM, CpG or anti-IgM/CpG and concomitantly treated with reserpine (1 μM) or NE (10 μM) for 1 h or left untreated. As control group, nonactivated B cells were used. The MFI of NS_521 (5 μM) was measured by flow cytometry. The MFI in relation to the control group is shown (*n* = 6). The gating strategy and representative histograms are included. (**B**, **C**) Anti-IgM/CpG-activated B cells were cultivated for 4 h or 24 h. Nonactivated B cells were used as control group. Immunofluorescence of cultivated B cells stained with NS_521 (10 μM; green) and Dapi (blue). Images were captured at 40× magnification with a Zeiss AxioVision microscope. Scale bars indicate 20 μm (main images) and 10 μm (insets). One of 3 representative images is shown (B; *n* = 3). The MFI of NS_521 in B cells was measured with the AxioVision software (C; *n* = 34 B cells/condition). (**D**) 1 × 10^7^ splenic B cells from DBA/1J mice were activated with anti-IgM/CpG (5 μg/ml / 1.25 μg/ml) for 4 h and 24 h or were left inactivated. M30 dihydrochloride (MAO inhibitor, 1 μM, Tocris, catalog number: 6067) and OR-486 (COMT inhibitor, 1 μM, Tocris, catalog number: 0483) was added to cultured B cells to reduce catecholamine degradation. After incubation, EDTA (1 mM) and sodium metabisulfite (4 mM) were supplemented to cell culture medium and B cell pellets to additionally prevent catecholamine degradation. B cells were lysed in water under ultra-sonification. Cell debris were centrifuged down at 1,500 × g for 10 min at 4°C. The concentration of dopamine, NE, and epinephrine was determined by competitive ELISAs in lysed B cells and in the supernatant of B cell cultures (*n* = 3–4). A medium control (supernatant group) and water control (B cells) was included in the figure. Statistical significance was determined by ordinary 1-way ANOVA followed by Dunnett multiple comparison test (A) or Student *t* test (C, D). n.s., not significant; *p < 0.5; **p < 0.01; ***p < 0.001; ****p < 0.0001. For underlying data, see S1 Data. ANOVA, analysis of variance; COMT, catechol-o-methyltransferase; EDTA, ethylenediaminetetraacetic acid; MAO, monoamine oxidase; MFI, median fluorescence intensity; NE, norepinephrine; NS_521, neurosensor_521.

Catecholamines are secreted by sympathetic nerve fibers, thereby affecting the function of other cells. Some immune cells produce their own catecholamines [43–45], but it is yet unknown whether this is a regulated process. To investigate whether the production of catecholamines depends on efficiency and persistence of activation, B cells were either activated with the BCR stimulus anti-IgM, the TLR9 agonist CpG, with both (BCR/TLR9) or were left untreated. Accumulation of intracellular catecholamines was visualized by using neurosensor_521 (NS_521), a fluorescent dye that specifically reacts with dopamine and NE. After 4 h of activation, B cells showed elevated levels of catecholamines compared to unstimulated B cells (Fig 2A). Additionally, activation of B cells with anti-IgM alone resulted in minimal production of catecholamines, while most catecholamines were produced upon activation with anti-IgM/CpG (Fig 2A). Treating B cells with reserpine, a specific, irreversible inhibitor of the vesicular monoamine transporter (VMAT) 1 and 2, causes a profound depletion of endogenous catecholamines in B cells (Fig 2A). Furthermore, treating B cells with NE, an agonist acting on α- and β-ADRs in a concentration-dependent manner [46], reduced endogenous catecholamines in the cell, similar to the inhibitor reserpine (Fig 2A). However, after long-term activation (24 h), intracellular staining showed lower levels of catecholamines, and, therefore, we hypothesized that catecholamines are released from the cell (Fig 2A). In addition to flow cytometry, immunofluorescence studies support our results that B cells up-regulate intracellular catecholamines after short-term incubation, whereas long-term stimulation seems to foster the release of produced catecholamines (Fig 2B and 2C). In order to analyze if catecholamines are actually released from B cells, the concentration of dopamine, NE, and epinephrine was determined in B cells and in the supernatant of B cell cultures 4 h and 24 h after activation. We found that all catecholamines studied were detectable in B cells after 4 h (Fig 2D) and were already partially released from the cell, while after long-term stimulation (24 h), the majority of NE and epinephrine was reduced in the B cells and detectable in the supernatant (Fig 2D). In conclusion, our results suggest that synergistic activation of B cells with anti-IgM/CpG leads to enhanced production of catecholamines, which are released by B cells over time.

### TLR9 activation induces TH expression in B cells

**Fig 3:**
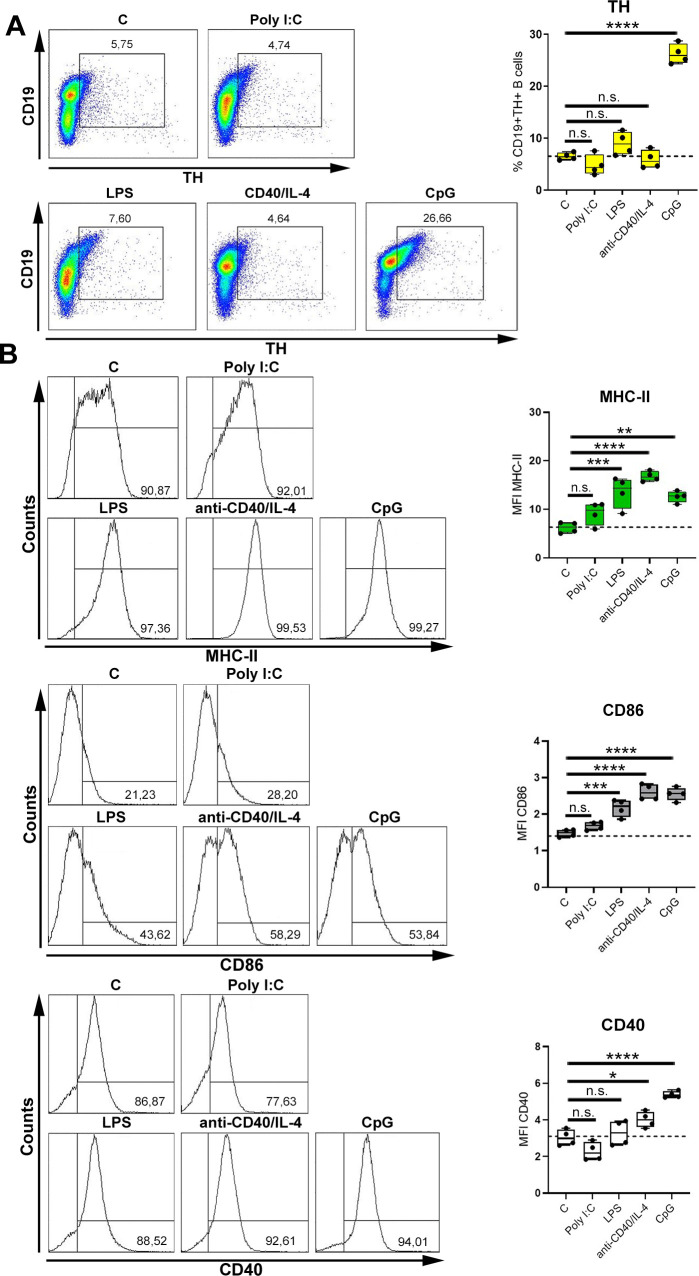
TLR9 activation induces TH expression in B cells. (**A, B**) B cells were activated with the TD stimulus anti-CD40 (1.25 μg/mL) in combination with IL-4 (1.25 ng/mL) or with the different TI-stimuli (Poly I:C (TLR3; 1.25 μg/mL), LPS (TLR4; 1.25 μg/mL) and CpG (TLR9; 1.25 μg/mL) for 24 h. As control group, nonactivated B cells were used. The expression of CD19+TH+ B cells (A; *n* = 4) and the MFI of MHC-II+, CD86+ and CD40+ B cells (B; *n* = 4) were quantified by flow cytometry. One representative dot plot (A) or histogram (B) is shown. B cells from naive DBA/1J mice were used for experiments. Statistical significance was determined by ordinary 1-way ANOVA followed by Dunnett multiple comparison test (A, B). n.s., not significant; *p < 0.5; **p < 0.01; ***p < 0.001; ****p < 0.0001. For underlying data, see S1 Data. ANOVA, analysis of variance; IL, interleukin; LPS, lipopolysaccharide; MFI, median fluorescence intensity; TD, T cell–dependent; TH, tyrosine hydroxylase; TI, T cell–independent; TLR, Toll-like receptor.

It is known that B cells are activated in a T cell–dependent (TD) or T cell–independent (TI) way by stimulation of CD40 or TLR signaling pathways, respectively. Therefore, we investigated whether up-regulation of TH in B cells is also mediated by other mechanisms than TLR9 activation. For this purpose, B cells were stimulated with the TD stimulus anti-CD40 in combination with IL-4 or ligands for TLR3, TLR4, and TLR9 (TI). Unstimulated B cells served as control. TLR9 activation was most effective in inducing TH (Fig 3A), albeit the TLR4 ligand lipopolysaccharide (LPS) and anti-CD40/IL-4 stimulation increased cell surface levels of major histocompatibility complex class II (MHC-II) and CD86 similar to the TLR9 ligand CpG (Fig 3B). Interestingly, higher concentrations of TD and TI mitogens resulted in increased expression of activation markers (S3A and S3B Fig) and TH expression (S4A Fig). Furthermore, by testing different CpG-oligodeoxynucleotides (ODNs), CpG-ODN 1826 was the strongest activation stimulus (S5A Fig), and this activation correlated with highest expression of TH (S5B Fig). Taken together, TH is increased in B cells upon TLR9 ligation.

### B cells express catecholamine receptors and transporters

**Fig 4:**
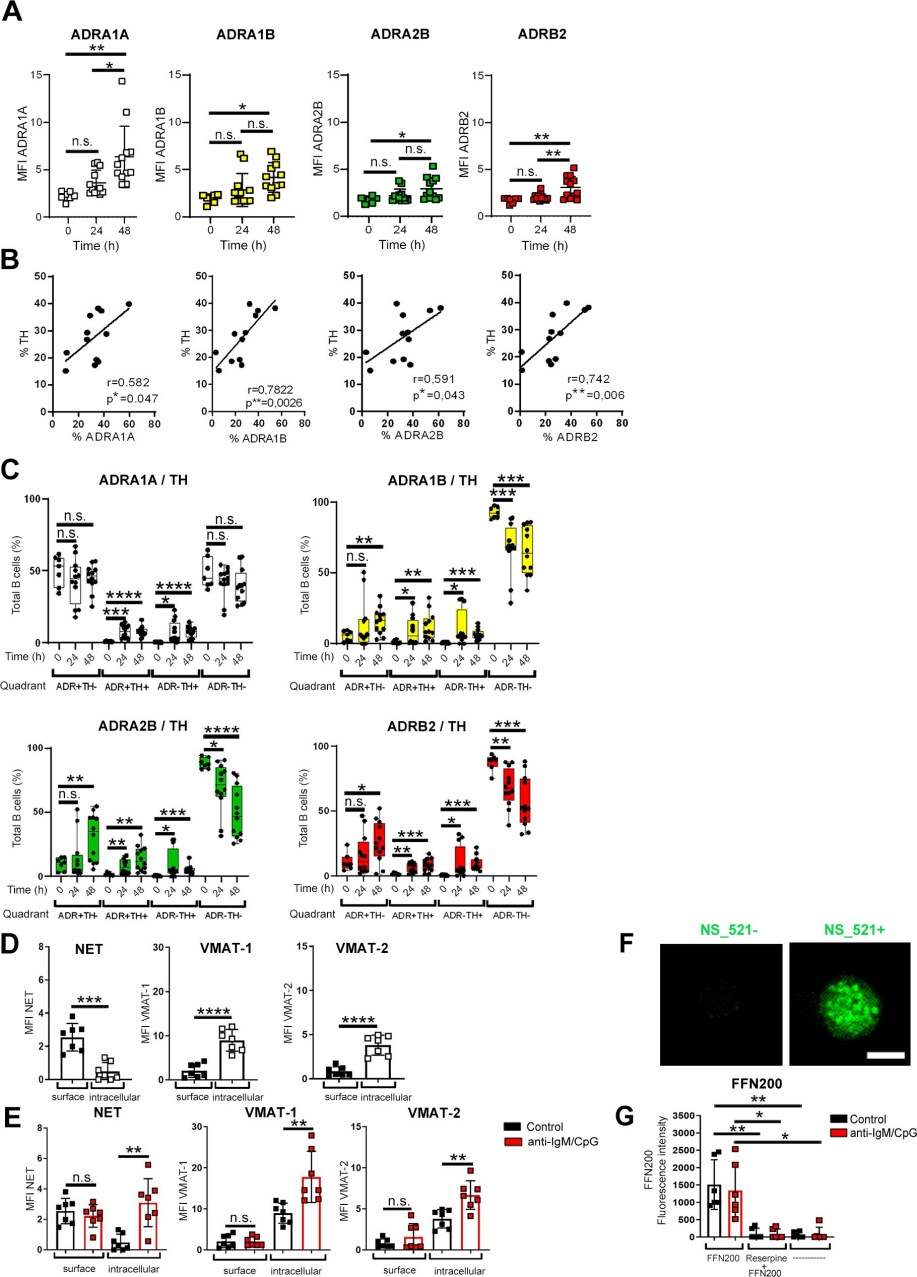
B cells express catecholamine receptors and transporters. (**A**–**C**) B cells were activated with anti-IgM/CpG for 24 h and 48 h. Nonactivated B cells were used as control group (0 h). (**A**) MFI of all ADRs analyzed (0 h: *n* = 7; 24 h, 48 h: *n* = 12) was determined by flow cytometry. The gating strategy is available in S6A Fig. (**B)** The correlation of TH and ADR expression was quantified after 48 h (*n* = 12). (**C**) The expression of different ADRs and TH expression (ADR+TH−, ADR+TH+, ADR−TH+, and ADR−TH−) was analyzed by flow cytometry (0 h: *n* = 7; 24, 48 h: *n* = 12). The gating strategy and representative dot plots of control group (0 h) and after activation (48 h) are shown in S6B Fig. (**D**, **E**) Nonactivated B cells (D) or anti-IgM/CpG-activated B cells (E) were cultivated for 24 h. The MFI of labeled monoamine transporters was determined by flow cytometry (*n* = 7). The gating strategy is available in S6C Fig. (**F**) Nonactivated B cells were stained with NS_521 (10 μM) for 30 min at 37°C and 5% CO_2_. Catecholamine-producing B cells were analyzed by immunofluorescence histology. Fluorescent images were captured at 40× magnification using Zeiss AxioVision microscope. One of 3 representative images is shown. Scale bar = 5 μm *(n* = 3). (**G**) Nonactivated and activated B cells were treated with reserpine for 45 min or left untreated. Afterward, the cells were incubated with FFN200 dihydrochloride (10 μM) for 1.15 h. FFN200 dihydrochloride- and reserpine-untreated cells were used as controls. The fluorescence intensity of FFN-200 dihydrochloride was measured by plate multimode reader (*n* = 6). For the experiments, B cells from naive DBA/1J mice were used. Data are pooled from 5 experiments (A–C). Statistical significance was determined by 1-way ANOVA followed by Tukey multiple comparison test (A), Brown–Forsythe, and Welch ANOVA tests followed by Dunnett T3 multiple comparison test (C, G), Student *t* test (D, E). Continuous variables were analyzed using linear regression with r values calculated by Pearson correlation (B). n.s., not significant; *p < 0.5; **p < 0.01; ***p < 0.001; ****p < 0.0001. For underlying data, see S1 Data. ADR, adrenergic receptor; ANOVA, analysis of variance; MFI, mean fluorescence intensity; NS_521, neurosensor_521; TH, tyrosine hydroxylase.

Catecholamines synthesized by B cells might act in an autocrine manner or are secreted to influence other cells by paracrine or endocrine mechanisms. Since the expression of ADRs is a prerequisite for stimulation with catecholamines, we determined which ADRs are expressed by B cells stimulated with anti-IgM/CpG. The surface expression of ADRA1A, ADRA1B, ADRA2B, and ADRB2 on B cells upon activation was determined and compared to nonactivated B cells. After 48 h, we found that activated B cells up-regulate all ADRs investigated as compared to unstimulated control cells (Fig 4A, S6A Fig), and this also correlated with enhanced expression of TH (Fig 4B). To further analyze whether the expression of TH coincides with the expression of ADRs, we analyzed anti-IgM/CpG-activated and nonactivated B cells for the expression of TH, ADRA1A, ADRA1B, ADRA2B, and ADRB2 (Fig 4C, S6B Fig) by flow cytometry. We found nonactivated B cells to be either ADR−TH+ (nonresponder and producer), ADR+TH− (responder and nonproducer), ADR+TH+ (responder and producer), or ADR−TH− (nonresponder and nonproducer). While activated B cells showed a similar expression pattern between the different ADRs and TH, a decrease in the proportion of ADR−TH− B cells and an increase in all other populations was observed (Fig 4C). These data suggest that TLR9-stimulated B cells are potentially modulated by their own monoamines in an autocrine and/or paracrine fashion. Monoamine transporters control the extracellular concentration of monoamine neurotransmitters. Therefore, we also tested whether B cells express catecholamine transporters and are able to regulate the uptake of catecholamines. The expression of 3 different transporters was investigated in B cells: the norepinephrine transporter (NET), VMAT-1, and VMAT-2. The latter are responsible for the shuttling of catecholamines into vesicles [47,48] while NET mediates the reuptake of catecholamines from extracellular space [49]. In unstimulated B cells, we detected NET on the plasma membrane, whereas VMAT-1 and VMAT2 were located intracellularly (Fig 4D, S6C Fig). In comparison to nonactivated B cells, activated B cells showed increased intracellular levels of all 3 transporters (Fig 4E). Moreover, intracellular catecholamines were visualized by treating nonstimulated B cells with the fluorescent catecholamine dye NS_521. The observed staining pattern suggested vesicular storage of catecholamines in B cells (Fig 4F), which is also suggested by decreased catecholamines following reserpine treatment, as described above. The functionality of transporters was assessed by incubating nonactivated and anti-IgM/CpG-activated B cells with a selective fluorescent VMAT2 substrate (FFN200 dihydrochloride) in the presence or absence of the H+-coupled VMAT blocker (reserpine). We confirmed the uptake of FFN200 by nonactivated and activated B cells (Fig 4G). Additional treatment with pan VMAT inhibitor reserpine blocked the uptake of FFN-200 resulting in reduced fluorescence intensity. In conclusion, we found that B cells have the prerequisite to respond to catecholamines via their ADRs and are able to transport and store catecholamines intracellularly for later release from the cell.

### TH up-regulation is associated with IL-10 production

**Fig 5:**
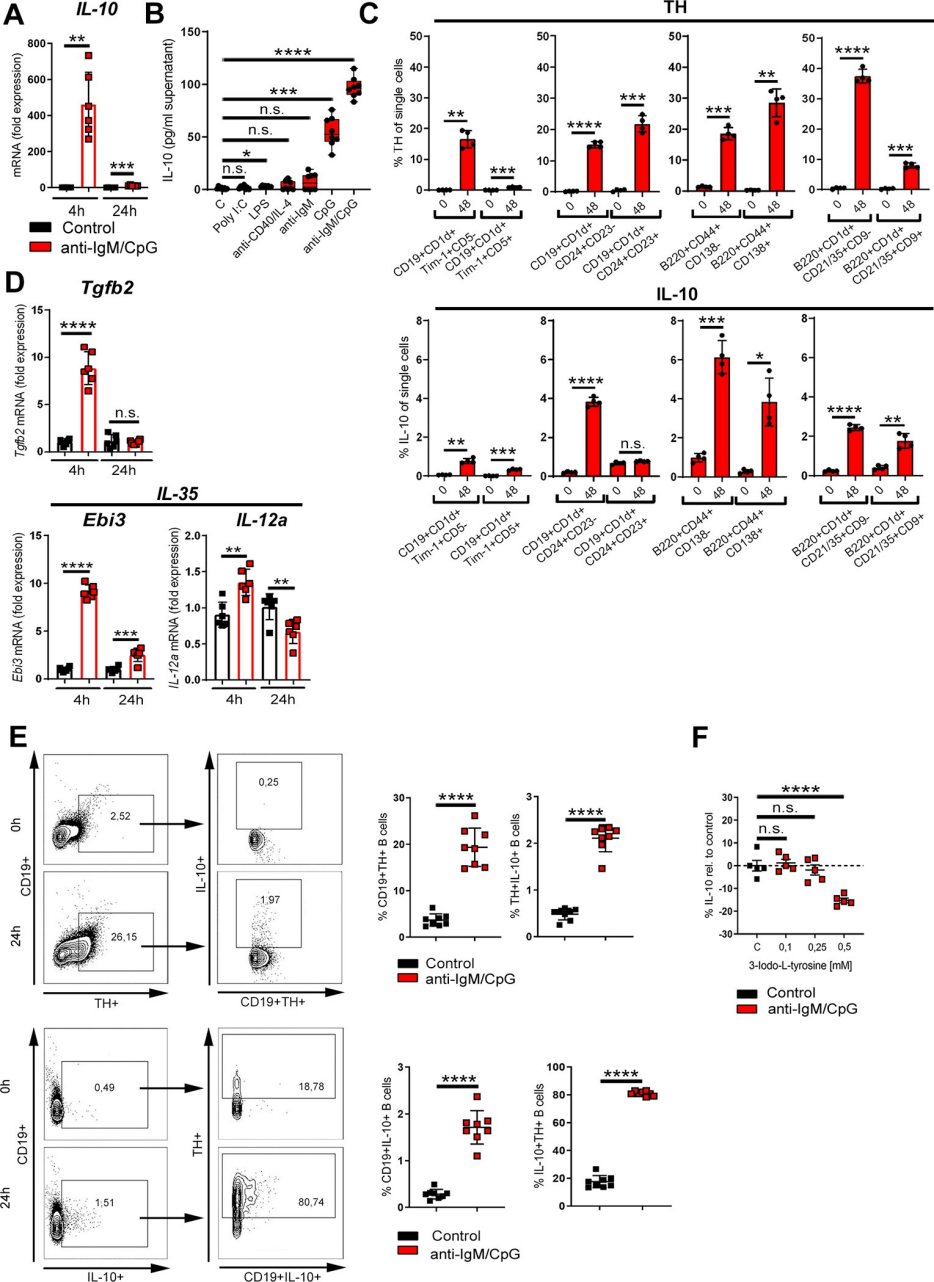
TH up-regulation is associated with IL-10 production. (**A**) B cells were activated for 4 h and 24 h with anti-IgM/CpG or left nonactivated. The expression of IL-10 was determined by qRT-PCR (*n* = 6). (**B**) B cells were activated with indicated TD or TI stimuli (anti-CD40; Poly I:C, LPS, CpG: 1.25 μg/mL; anti-IgM: 5 μg/mL; IL-4: 1.25ng/mL). Nonactivated B cells were used as controls. The production of IL-10 was measured in the cell culture supernatant by ELISA (*n* = 8). (**C**) B cells were activated with anti-IgM/CpG or left untreated. After 48 h, Breg populations and their TH and IL-10 expression were analyzed by flow cytometry and compared to control B cells (0 h) (*n* = 4). The gating strategy of indicated Breg populations with their characteristic surface markers is available in S8 and S9 Figs. (**D**) Anti-IgM/CpG-activated B cells or nonactivated cells were cultivated for 4 h or 24 h. The expression of indicated genes was determined by qRT-PCR (*n* = 6). (**E**) B cells were activated with anti-IgM/CpG for 24 h or left nonactivated. The expression of CD19+TH+, CD19+IL-10+, TH+IL-10+, and IL10+TH+ B cells was analyzed by flow cytometry (*n* = 8). One representative dot plot is shown. (**F**) B cells were treated with different concentrations of the TH inhibitor 3-Iodo-L-tyrosine for 30 min, before activation with anti-IgM/CpG. The production of IL-10 was analyzed in cell supernatants by ELISA (*n* = 5). B cells from naive DBA/1J mice were used for the experiments. Data are pooled from 3 (E) or 4 (B) experiments. Statistical significance was determined by Student *t* test (C, E) with Welch correction (A, D) or Brown–Forsythe and Welch 1-way ANOVA tests (B) or ordinary 1-way ANOVA followed by Dunnett multiple comparison test (F). n.s., not significant; *p < 0.5; **p < 0.01; ***p < 0.001; ****p < 0.0001. For underlying data, see S1 Data. ANOVA, analysis of variance; ELISA, enzyme-linked immunosorbent assay; IL, interleukin; LPS, lipopolysaccharide; Poly I:C, polyinosinic:polycytidylic acid; qRT-PCR, quantitative real-time polymerase chain reaction; TD, T cell–dependent; TH, tyrosine hydroxylase; TI, T cell–independent.

In previous studies, we found ex vivo ß2-ADR stimulation to augment B cell–derived IL-10 production [4]. In this study, we confirmed that activation with anti-IgM/CpG induced IL-10 expression in B cells from DBA/1J mice as assessed by qRT-PCR (Fig 5A), ELISA (Fig 5B, S7B Fig), and flow cytometry (S7A Fig). The B cell–induced IL-10 expression was comparable to C57BL/6 and BALB/c mice (S2B Fig). In line with our finding that CpG increased levels of TH, we also confirmed that among different classes of CpG-ODNs, the CpG-ODN 1826 enhanced IL-10 production (S5C Fig). Higher concentrations of TD (anti-CD40/IL-4) and TI mitogens (LPS, Poly I:C) were also able to induce IL-10 production (S4B Fig), but levels were lower as compared to CpG activation (Fig 5B). Therefore, CpG not only increases TH expression but also induces a regulatory, IL-10–positive B cell phenotype, which was confirmed by flow cytometry showing an increased percentage of distinct types of Breg populations and their TH expression after TLR9 stimulation (Fig 5C, S8 and S9 Figs). Furthermore, expression of TGF-β and IL-35, which are also markers for Bregs, was enhanced (Fig 5D). Next, we investigated whether expression of TH is associated with IL-10 production in B cells by flow cytometry. Almost 80% of IL10+ B cells were TH positive (Fig 5E), showing a strong association of catecholamine-producing capability and regulatory function. Moreover, inhibition of TH by using the specific TH inhibitor 3-Iodo-L-tyrosine resulted in a significant reduction of IL-10 production by B cells (Fig 5F), while B cell viability was not affected by this treatment (S10 Fig). From these data, we conclude that TLR9 agonism leads to intrinsic TH up-regulation and differentiation of B cells into distinct types of Breg populations with a concomitant increase in IL-10 production.

### Activation of ß-ADRs enhance B cell–derived IL-10 production

**Fig 6:**
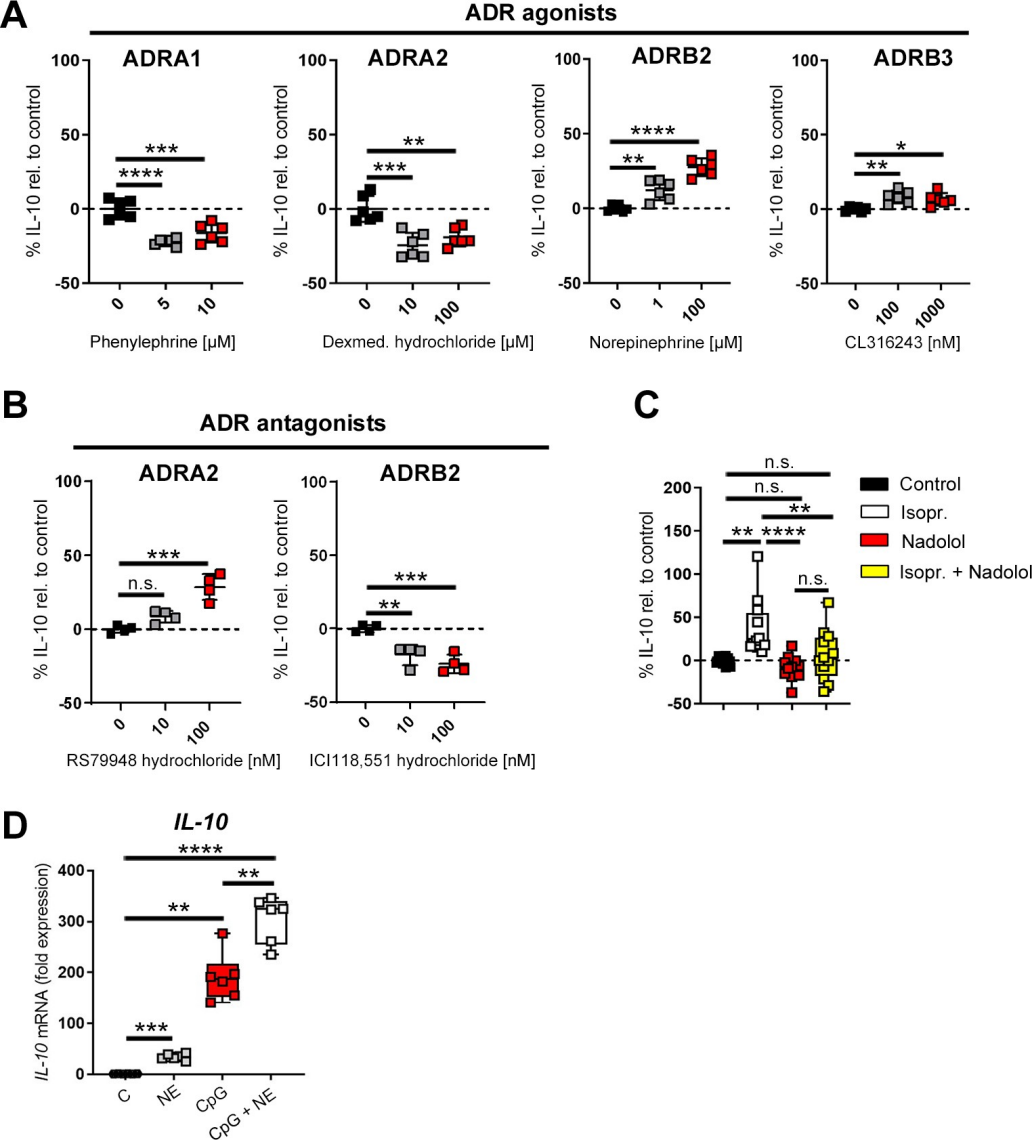
Activation of ß-ADRs enhance B cell–derived IL-10 production. (**A**) Anti-IgM/CpG-activated or nonactivated B cells were cultivated for 4 h and additionally treated with different concentrations of α1-, α2- or β-ADR agonists for 24 h (*n* = 6). (**B**) B cells were either treated with the indicated concentrations of the α2-ADR antagonist RS79948 hydrochloride or with the ß2-ADR antagonist ICI118,551 30 min before activation with anti-IgM/CpG for 24 h (*n* = 4). (**C**) B cells were either treated with the β-ADR antagonist nadolol [5μM] 30 min before activation with anti-IgM/CpG or were at first activated with anti-IgM/CpG for 4 h before treating cells with the pan ß-ADR agonist Isopr. (10 μM) (*n* = 12). The production of IL-10 was analyzed by ELISA (A–C). (**D**) B cells were cultivated with NE (10 μM), CpG (1.25 μg/ml) or both (CpG+NE) for 4 h. Nontreated B cells were used as control group. The IL-10 mRNA expression was analyzed by qRT-PCR (*n* = 6). B cells from naive DBA/1J mice were used for the experiments. Data are pooled from 3 (D) or 5 experiments (C). Statistical significance was determined by ordinary 1-way ANOVA followed by Dunnett multiple comparison test (A, B) or Tukey multiple comparison test (C) or Brown–Forsythe and Welch ANOVA test followed by Tamhane T2 multiple comparison test (D). n.s., not significant; *p < 0.5; **p < 0.01; ***p < 0.001; ****p < 0.0001. For underlying data, see S1 Data. ADR, adrenergic receptor; ANOVA, analysis of variance; ELISA, enzyme-linked immunosorbent assay; IL, interleukin; Isopr., isoproterenol; NE, norepinephrine; qRT-PCR, quantitative real-time polymerase chain reaction.

Since anti-IgM/CpG-activated B cells up-regulate several ADRs, we checked whether ADR signaling is important for acquiring a regulatory phenotype. For this purpose, anti-IgM/CpG-primed B cells were treated with agonists for α1-, α2-, β2-, and β3-ADRs or with antagonists for α2- and β2-ADRs. Activation of α1- and α2-ADRs led to a significant and concentration-dependent reduction in IL-10 production (Fig 6A), while blocking of α2-ADRs elevated IL-10 levels (Fig 6B). By contrast, stimulation of B cells with subtype-specific β-ADR agonists enhanced IL-10 production (Fig 6A), while inhibition of ß2-ADRs reduced IL-10 levels (Fig 6B). Moreover, simultaneously stimulation of all different ß-ADRs with the nonselective ß-ADR agonist isoproterenol additionally enhanced IL-10 production (Fig 6C). This effect was inhibited by the pan-ß-ADR antagonist nadolol (Fig 6C). Moreover, ß-ADR stimulation fostered IL-10 production independently of CpG activation and significantly enhanced CpG-induced IL-10 levels (Fig 6D). These data show that B cell–derived IL-10 production is determined by integration of ß-ADR and α-ADR signals and therefore modulated dependent on catecholamine concentration in the vicinity of the B cell.

### IL-10 improves Breg function to suppress T cells

**Fig 7:**
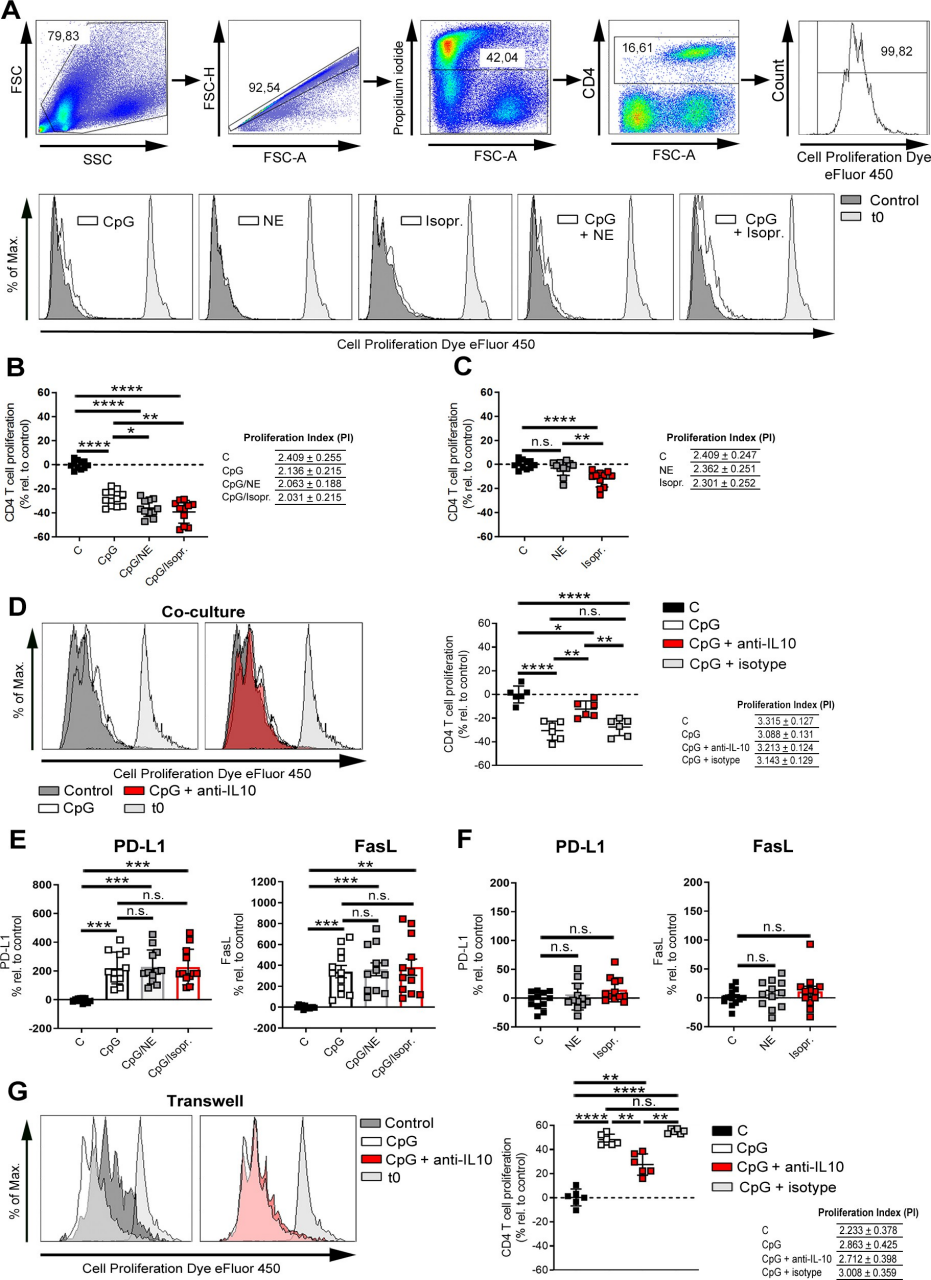
IL-10 improves Breg function to suppress T cells. (**A**–**G**) DBA/1J mice were immunized with 100 μl of an emulsion of CII and CFA. After 14–21 days, splenic B cells from immunized mice were isolated by MACS and B cell–depleted splenocytes were used for coculture and transwell experiments. B cells were preactivated with CpG (1.25 μg/mL), Isopr. (10 μM), or NE (10 μM) alone or in combination or left untreated. Splenocytes were labeled with the cell proliferation dye eFluor 450 and activated with CD3/CD28 before co-cultivation with B cells for 72 h. (**D**) Moreover, the coculture of CpG-preactivated B cells and CD3/CD28-activated splenocytes was additionally incubated with or without an anti-IL-10 depletion or anti-IgG isotype control antibody. The proliferation (A, B, C, and D) of CD4+ T cells was monitored by flow cytometry (B, C: *n* = 11; D: *n* = 6). One representative histogram is shown (A, D). (**E, F**) The expression of PD-L1 and FasL was analyzed on cocultured B cells after 72 h by flow cytometry (PDL-1, FasL: *n* = 12). (**G**) CpG-preactivated B cells and CD3/CD28-activated splenocytes were separately cultivated by using transwells and additionally treated with or without an anti-IL-10 depletion or anti-IgG isotype control antibody. After 72 h, the proliferation of CD4+ T cells was analyzed by flow cytometry (*n* = 6). Data are pooled from 4 experiments (B, C, E, and F). The PI has been calculated by using the number of events measured in each division peak. Statistical significance was determined by ordinary 1-way ANOVA (B–D, F) or Brown–Forsythe and Welch ANOVA followed by Tamhane T2 multiple comparison test (E, G). n.s., not significant; *p < 0.5; **p < 0.01; ***p < 0.001; ****p < 0.0001). For underlying data, see S1 Data. ANOVA, analysis of variance; Breg, regulatory B cell; CFA, complete Freund’s adjuvant; CII, type II collagen; FasL, Fas ligand; IL, interleukin; Isopr., isoproterenol; MACS, magnetic-activated cell sorting; NE, norepinephrine; PD-L1, programmed death-ligand 1; PI, proliferation index.

Bregs inhibit or suppress inflammation mediated by CD4+ T cells, mainly T helper 1 cells (Th1) [50,51], which is one of the main populations that plays a crucial role in the pathogenesis of auto-inflammatory diseases like RA. In order to demonstrate the development of antigen-primed Bregs during CIA, B cells from mice with CIA were pulsed with type II collagen (CII) antigen or peptide before co-culturing with CD3/CD28-activated and eFluor450-labeled CD4+-containing splenocytes. We found that CII-antigen-pulsed B cells but not CII-peptide-primed B cells suppressed the proliferation of CD4 T cells in a concentration-dependent manner (S11 Fig). However, Bregs generated by TLR9 activation with or without concomitant ß-ADR stimulation are also able to inhibit the proliferation of CD4+ T cells in a similar manner (Fig 7A and 7B). Moreover, ß-ADR stimulation of CpG preactivated B cells further enhanced CD4+ T cell suppression (Fig 7A and 7B). Even isoproterenol alone slightly suppressed T cell proliferation independent of CpG-activation (Fig 7A–7C). These results suggest that CpG-primed Bregs have the highest capacity to suppress inflammation induced by CD4+ T cells, which is additionally enhanced by a ß-ADR stimulus (Fig 7A and 7B). Several mechanisms are responsible for Breg-mediated immunosuppression. To determine the role of IL-10 in CpG-induced CD4+ T cell suppression, anti-IL-10 depleting antibodies were added to coculture medium, resulting in decreased suppression of CD4+ T cells in comparison to isotype control antibodies (Fig 7D). Furthermore, to analyze if the enhanced suppression of CD4+ T cell proliferation by a ß-ADR stimulus is mediated via IL-10, wild-type (WT) and IL-10^−/−^ mice were used. IL-10^−/−^ mice failed to enhance the suppression of CD4 T cell proliferation by an additional ß-ADR stimulus compared to WT mice (S12 Fig), suggesting that the enhanced suppression of T cell proliferation following ß-ADR stimulation is mediated by IL-10. However, as compared to WT mice, basal level of suppression was already higher after CpG treatment, suggesting compensatory mechanisms in IL-10^−/−^ B cells. In addition to IL-10, Bregs impair T cell function via surface proteins such as PD-L1 [37,38] and FasL [39,40]. Therefore, we determined whether Bregs generated by TLR9 activation show enhanced expression of these proteins. Indeed, we demonstrated that both PD-L1 and FasL were significantly up-regulated on TLR9-activated B cells in comparison to untreated cells (Fig 7E). However, the expression levels were not dependent on ß-ADR stimulation (Fig 7E and 7F). In order to assess the impact of cell–cell interaction, cytokine production, or both in the suppression of CD4+ T cell proliferation, trans-well experiments were performed. However, in this setting, TLR9-activated B cells promoted CD4+ T cell proliferation rather than to suppress it (Fig 7G). This increased proliferation was due to the presence of IL-10, because depletion of IL-10 significantly reduced CD4+ T cell proliferation (Fig 7G). In conclusion, TLR9-activated B cells primarily suppress CD4+ T cell proliferation in a cell–cell contact–dependent way, and this suppression is significantly enhanced by additional presence of IL-10 and ß-ADR signaling.

## Discussion

In this study, we report that TLR9-activated B cells not only express increased levels of catecholamine-producing enzymes, like TH and PNMT, but also have the ability to synthesize their own catecholamines in a time- and stimulus-dependent manner. Moreover, they employ endogenous catecholamines for autocrine and/or paracrine signaling to influence their function and possess α- and β-ADRs as well as monoamine transporters, which control uptake and storage of catecholamines. Furthermore, IL-10 production is associated and modulated by TH and the ability of TLR9-Bregs to suppress CD4+ T cells is primarily mediated by increased expression of co-inhibitory surface molecules like, e.g., PD-L1 and FasL, but additionally enhanced by elevated IL-10 production via ß-ADR stimulation.

The interaction between the nervous system and immune system through sympathetic nerve fibers is of high importance. Disturbance of this interaction was shown to be involved in several immune system disorders, not only during viral infection [52], but also in autoimmune diseases [53–55]. Among others, B cells are a target of the SNS, and several studies have shown an important physiological role of this interaction, e.g., in the context of specific and polyclonal antibody production [9,56,57]. An additional backup strategy should exist to recover the lack of brain control upon loss of peripheral sympathetic nerves during chronic inflammation [10,41]. The ability of B cells to compensate the decreased adrenergic signaling might be considered as one of the checkpoint mechanisms to locally prevent hyperactivation of the immune system and immunopathology.

We identified that B cells are functionally able to compensate the loss of sympathetic nerve fibers in chronically inflamed tissue, since they possess proteins necessary to synthesize, sense and transport catecholamines resulting in suppression of CD4+ T cell proliferation. Whether B cells alone can take over catecholaminergic signaling from sympathetic nerves lost in chronically inflamed joints and tissues or need support of other cells has to be investigated in further studies. In this context, questions arise about the amount of catecholamines produced by B cells and about the concentration needed to fully compensate the loss of sympathetic nerve fibers. However, we show that blocking TH results in decreased IL-10 production in a pure B cell culture underscoring the importance of B cell catecholamine production for B cell regulatory function. We determined the concentration of dopamine, NE, and adrenaline in B cells and in the supernatant of B cell cultures. Short-term activation of B cells increased the concentration of all investigated catecholamines in B cells and its partial release from the cell. While long-term activation showed a reduced concentration of catecholamines in B cells and elevated amounts in the supernatant. This is evidence that B cells synthesize dopamine, NE, and epinephrine and subsequently release these catecholamines from the cell. Medium control shows higher dopamine concentrations as compared to supernatant taken from activated and nonactivated cells, which can be interpreted as indication for the ability of B cells to take up catecholamines from the medium. To our knowledge, this is the first study to comprehensively characterize catecholamine metabolism in B cells.

NS_521 was used to directly detect and monitor catecholamine content in B cells. With this fluorescent dye, we demonstrated that catecholamines are detectable in B cells by flow cytometry and that they are stored in synaptic-like vesicles by a VMAT-mediated uptake mechanism, thus mimicking further functional aspects of neuronal tissue. Monitoring of catecholamine content was previously only performed in chromaffin cells characterized by HPLC [58,59] or capillary electrophoresis assays with electrochemical detection [43,44]. To our knowledge, this is the first time to directly observe catecholamine metabolism in immune cells.

ADRs are also expressed on other immune cells such as T cells [60]. Different studies showed that signaling through these receptors is important to regulate their function and an impaired signaling of β2-ADR in circulating T cells promotes immunological diseases such as RA [16,18,61]. It remains to be determined whether T cells are also influenced in their regulatory function by catecholamines produced by B cells. Some authors have proposed that also T cells express TH [62, 63], and similar mechanisms as described for B cells in the present report might also be valid for T cell or other TH expressing immune cells. However, there are also immune cells, like macrophages, which do express transporters for catecholamines, enabling them to take up catecholamines but not synthesizing them on their own [64]. Overall, these findings argue for a differentiated and independent sympathetic system throughout the immune system, which is not necessarily controlled by higher level systems like the CNS.

Bregs influence various immune cells, for example, by inhibiting the differentiation of Th1 and Th17 cells [50, 1] or by affecting cytokine production of monocytes and dendritic cells [50,65], while promoting the expansion of regulatory T cells [66–68]. In coculture experiments, we found that TLR9-activated Bregs are able to inhibit the proliferation of CD3/CD28-activated CD4+ T cells via the expression of inhibitory cell–cell contact molecules, supported by IL-10. The strong up-regulation of PD-L1 and FasL expression on B cells after TLR9 activation suggests that these inhibitory molecules are crucial to inhibit CD4+ T cell proliferation. Furthermore, we demonstrated that Breg-mediated suppression through cell surface molecules was further enhanced by the production of IL-10 and ß-ADR signaling. Separation of activated B cells and splenocytes through a membrane (transwell) did not lead to suppression of CD4 T cells, but vice versa increased T cell proliferation. This indicates that soluble factors such as IL-10 are not self-sufficient to directly augment T cell suppression but enhance cell surface signals. This hypothesis is supported by a previous study demonstrating that treatment of activated T cells with different concentrations of recombinant IL-10 does not directly inhibit proliferation, but first affects antigen-presenting cells (APCs), which then suppress T cells [69]. Moreover, the modulating effect of IL-10 on B cells was already demonstrated in earlier studies [70,71]. Whether IL-10 influences the expression of cell inhibitory molecules, e.g., PD-L1, FasL, to increase cell contact dependent suppressive activity of B cells directly [38,40,72–74] is currently unknown but is suggested by previous data showing IL-10-inducible PD-L1 expression on monocytes [75,76]. Another interpretation of our data is that IL-10 directly affects T cell suppression, but only in the presence of signaling from cell inhibitory molecules.

In several experimental mouse models of autoimmune diseases, a reduced Breg function has been observed [77]. This is in line with studies in humans, showing a reduction or functional impairment of IL-10-producing Bregs in active autoimmune diseases like systemic sclerosis [78,79], ANCA-associated vasculitis [80,81], RA [82–84], or systemic lupus erythematosus [73]. Observations show that Breg numbers return to normal levels when patients are in remission. These observations suggest that augmenting Breg function or number *ex vivo* and transferring these cells back into the host ameliorates disease and restores the balance between pro-inflammatory and anti-inflammatory signals. Indeed, in some experimental mouse models, it has been validated that transfer of Bregs back into the host improves several autoimmune diseases including CIA [77,85], experimental autoimmune encephalomyelitis (EAE) [86], or lupus [31]. A positive role of the SNS in this context is supported by previous experiments showing that only transfer of splenic B cells from mice with an intact SNS and not B cells from sympathectomized mice ameliorates CIA [4].

In the present study, we discovered that more than 80% of IL-10-producing B cells also express TH. A detailed analysis of CpG-induced Bregs revealed the development of distinct TH+ and IL-10 expressing Breg populations, which have been identified as marginal zone B cells (B220+ CD1d+CD21/35+CD9+), plasmablasts (B220+CD138+CD44hi), T2-MZP B cells (CD19+CD1hiCD23hiCD24hi), and CD1d+Tim-1+CD5+ B cells. Due to the increase of TH in activated B cells, we suggest TH as a general marker for at least TLR9-activated B cells. If TH can be also classified as an additional biomarker for Bregs further studies are needed to get information about its long-term expression in Bregs.

However, a previous study confirmed that there is a general connection between TH expression and the anti-inflammatory properties of cells, since adoptive transfer of in vitro generated TH+-dependent anti-inflammatory neuronal cells leads to significant reduction of CIA in mice [87]. Although the exact mechanism by which TH expression leads to amelioration of disease was not reported, the authors hypothesized that catecholamine production is important. These findings are in line with our results, since activated B cells increase TH after TLR9 activation and, during course of CIA, produce catecholamines and augment suppressive effects via increased IL-10. However, our results also show that the influence of catecholamines on B cell function depends on the pattern of ADR subtypes expressed and therefore is not static but might depend on context factors that determine these patterns. While additional stimulation via ß-ADRs significantly improved Breg function, activation via α-ADRs reduced IL-10 production. That ß-ADR signaling pathways play an important role in increasing IL-10 is supported by other recent publications [88,89]. In this context, we demonstrated that simultaneous activation of all ß-ADR subtypes, not only β2-ADR, synergistically augments IL-10 production, while blocking of ß-ADRs or stimulating α-ADRs reduced the levels of IL-10. These results indicate that there is a strong link between the amount of catecholamines and the anti-inflammatory potential of B cells, since, e.g., NE preferentially binds to α-ADR at low and ß-ADR at high concentrations [46]. The findings may be helpful in the development of new therapeutic strategies, like the ex vivo generation of Bregs and subsequent autologous transfer or by including ß-ADR agonists or α-ADR antagonists in current therapeutic regimens to increase Breg function in autoimmunity.

In this study, we showed for the first time that TLR9-activated B cells, similar to neurons, are able to synthesize, store, and release catecholamines. In addition, B cells take up and respond to catecholamines, which are important for modulating Breg function in an autocrine and/or paracrine manner by functional ß- and α-ADRs. Furthermore, we contributed to the general understanding of Breg function by presenting data that show that IL-10 is not self-sufficient to promote T cell immunosuppression and rather augments cell contact–dependent inhibitory mechanisms. In addition, we suggest that TH might be considered as a general marker for activated B cells. Taken together, this intriguing, self-sustained catecholamine system in B cells but also other immune cells might be exploited to develop new treatment strategies for immune-mediated diseases.

## Materials and methods

### Mice

All experiments were performed with animals housed in single ventilated cages, under the authorization of the Veterinäramt Nordrhein Westfalen (Düsseldorf, Germany) and in accordance with the German law for animal protection. Male DBA/1J mice, 6 to 10 weeks old, were originally purchased from Janvier Labs (Roubaix, France). C57BL/6 and BALB/c mice were obtained from the ZETT (Düsseldorf, Germany). IL-10^−/−^ mice were obtained from Prof. J.G. Bode and Dr. C. Ehlting (Clinic for Gastroenterology, Hepatology and Infectiology, University Hospital Düsseldorf, Germany). Animals were housed 5 in each cage and were fed standard laboratory chow and water ad libitum under standard conditions of temperature and light. Animals care was in accordance with institutional guidelines. All experimental protocols were approved by the Landesamt für Natur, Umwelt und Verbraucherschutz (LANUV) Nordrhein-Westfalen.

### CIA

Male DBA/1J mice (8 to 10 weeks old) were intradermally immunized at the base of their tails with 100-μl emulsion containing bovine CII (2 mg/ml, Chondrex, Redmond, Washington, United States of America) emulsified in an equal volume of complete Freund’s adjuvant (CFA; 2mg/mL, Chondrex) to generate CIA. Preparation of the emulsion was done according to the manufacturer’s protocol. Mice were used at the indicated time points after the initial injection.

### Antibodies

#### Primary antibodies

Anti-Alpha-1A adrenergic receptor (ADRA1A, Abcam, Cambridge, United Kingdom, Clone: EPR9691(B)); anti-Alpha-1B adrenergic receptor (ADRA1B, Abcam, Clone: EPR 10336); anti-Alpha-2B adrenergic receptor (ADRA2B, Abcam, Clone: EPR9623); anti-Beta-2 adrenergic receptor (ADRB2, Abcam, Clone: EPR707(N)); anti-CD1d-PE-Vio770 (Miltenyi Biotec, Bergisch Gladbach, Germany, Clone: 1B1); anti-CD4-APC-Vio770 (Miltenyi Biotec, Clone: REA604); anti-CD5-PerCp-Vio700 (Miltenyi Biotec, Clone: REA421); anti-CD9-APC (Miltenyi Biotec, Clone: MZ3); anti-CD19 (APC, PE, VioBright-Fitc, Miltenyi Biotec, Clone: 6D5); anti-CD21/35-VioBright B515 (Miltenyi Biotec, Clone: REA800); anti-CD23-APC (Miltenyi Biotec, Clone: REA1068); anti-CD24-APC-Vio770 (Miltenyi Biotec, Clone: REA743); anti-CD40-APC (Miltenyi Biotec, Clone: FGK45.5); anti-CD44-VioBright Fitc (Miltenyi Biotec, Clone: REA644); anti-CD45R (B220)-APC-Vio770 (Miltenyi Biotec, Clone: RA3-6B2); anti-CD86-APC-Vio770 (Miltenyi Biotec, Clone: PO3.3); anti-CD138-APC (Miltenyi Biotec, Clone: REA104); anti-Fas Ligand-APC (FasL, CD178, eBioscience, Thermo Fisher Scientific, Clone: MFL3); anti-Interleukin-10 (IL-10-PE, Miltenyi-Biotec, Clone: JES5-16E3); anti-Interleukin-10 (R&D Systems, Minneapolis, Minnesota, USA; Clone: JES052A5) for depletion; anti-Major Histocompatibility Complex II (MHC-II-PE (Miltenyi Biotec, Clone: REA610); anti-Norepinephrine transporter (NET, Alomone, catalog number: AMT-002); anti-Programmed death ligand 1 (PD-L1-PE/Cy7, BioLegend, London, UK, Clone: 10F.9G2); anti-Tim-1-APC (Miltenyi Biotec, Clone: REA692); anti-Tyrosine hydroxylase-Alexa Fluor488 and anti-Tyrosine hydroxylase-PE (TH, FACS: Abcam, Clone: EP1533Y); anti-vesicular monoamine transporter-1 (VMAT-1, Alomone, catalog number: AMT-007); and anti-vesicular monoamine transporter-2 (VMAT-2, Alomone; catalog number: AMT-006).

#### Secondary antibody

Goat anti-rabbit IgG H&L-Alexa Fluor 488 (Abcam, catalog number: ab150077).

#### Isotype controls

Rabbit IgG-PE and rabbit IgG-Alexa Fluor 488, monoclonal (Abcam, Clone: EPR25A); rabbit IgG, monoclonal (Abcam, Clone: EPR25A); rabbit IgG, polyclonal (Abcam, catalog number: ab37415); rat IgG kappa (eBioscience, Thermo Fisher Scientific, Clone: eBRG1); rat IgG 2bk-PE/Cy7 (BioLegend, Clone: RTK4530); armenian hamster IgG-APC (eBioscience, Thermo Fisher Scientific, Clone: eBio299Arm); rat IgG2ak (APC, APC-Vio770, PE, PerCpVio700, VioBright-Fitc, Miltenyi Biotec, Clone: ES26-15B7.3); rat IgG2bk (APC-Vio770, PE, PE-Vio770, Miltenyi Biotec, Clone: ES26-5E12.4); and REA control (APC, APC-Vio770 PE, VioBright Fitc, VioBright B515; Miltenyi Biotec, Clone: REA293).

### B cell isolation

Splenic mouse B cells from naive or immunized DBA/1J mice were isolated by magnetic-activated cell sorting (MACS) via negative selection with the mouse B cell isolation Kit (Miltenyi Biotech, catalog number: 130-090-862) according to the manufacturer’s protocol. The purity was controlled by flow cytometry and was 98%. B cell–depleted splenocytes were used for T cell suppression assay.

### Cultivation of B cells

Splenic mouse B lymphocytes were cultivated in Roswell Park Memorial Institute (RPMI) 1640 medium (Gibco, Thermo Fisher Scientific, Paisley, UK) containing GlutaMax (200 mM L-alanyl-L-glutamine-dipeptide), 25 mM HEPES (2(-4-(2-hydroxyethyl)-1-piperanzinyl) ethansulfonacid) and supplemented with 10% (v/v) fetal bovine serum (FBS; Gibco, Thermo Fisher Scientific), 10,000 U/mL (v/v) Penicillin-Streptomycin (Gibco, Thermo Fisher Scientific), and 0.1% (v/v) 2-mercaptoethanol (ß-ME, Merck, Darmstadt, Germany). B cell cultures were maintained by 37°C containing 5% CO_2_.

### Activation of B cells

B cells were treated with different concentrations of TI mitogens LPS (Sigma-Aldrich, Steinheim, Germany, catalog number: L2630), Polyinosinic-polycytidylic acid sodium salt (Poly I:C, Sigma, catalog number: P1530), and CpGs (mouse TLR9 agonist kit, InvivoGen, catalog number: tlrl-kit9m) or TD mitogen anti-CD40 (Invitrogen, Carlsbad, California, USA; Clone: 1C10) in combination with or without recombinant interleukin-4 (IL-4, Miltenyi Biotec, catalog number: 130-094-061) for 24 h. After testing the different CpG-ODNs in S5 Fig, CpG-ODN 1826 (1.25 μg/ml, InvivoGen, catalog code: tlrl-1826-1) was used for all further experiments in this study. For BCR activation, cells were treated with 5 μg/mL anti-IgM F(ab’)2(μ chain) fragment (Jackson ImmunoResearch, West Grove, Pennsylvania, catalog number: 115-006-075).

### Treatment of B cells with different ADR agonists and antagonists

For in vitro stimulation with ADR ligands, splenic B cells were seeded at 2.5 × 10^5^ cells per well in a 96-well plate. B cells were either incubated with the pan ß-ADR antagonist nadolol [5 μM, Sigma-Aldrich] or the indicated concentrations of α-and ß-ADR antagonists 30 min prior to activation followed by treatment with 5 μg/mL anti-IgM and 1.25 μg/mL CpG or were first activated with anti-IgM/CpG for 4 h followed by incubation with indicated concentrations of α- or ß-ADR agonists for 24 h. (**Agonists**: ADRA1: (R)-(-)-Phenylephrine hydrochloride (Tocris, Bristol, UK, catalog number: 2838), ADRA2: Dexmedetomidine hydrochloride (Tocris, catalog number: 2749), ADRB2: L-(-)—NE (+)-biartrate salt monohydrate (Sigma-Aldrich, catalog number: A9512), ADRB3: CL316243 disodium salt (Tocris, catalog number: 1499), ADRB: (-)-Isoproterenol hydrochloride (Sigma-Aldrich, catalog number: I6504). **Antagonists**: ADRA2: RS79948 hydrochloride (Tocris, catalog number: 0987), ADRB2: ICI 118,551 hydrochloride (Tocris, catalog number: 0821).

### Functionality of monoamine transporter VMAT-1/2

To test the functionality of the catecholamine transporters VMAT-1/2, anti-IgM/CpG-activated or nonactivated B cells were incubated with or without reserpine (5 μM, Tocris, catalog number: 2747), an inhibitor of VMAT-1 & VMAT-2 for 45 min. Then, B cells were incubated with FFN200 (10 μM, Tocris, catalog number: 5911), a selective substrate for the VMAT-2 for 1.15 h. Fluorescence intensity was quantified in the multimode plate reader Infinite 200 PRO (Tecan, Männedorf, Switzerland).

### Total RNA extraction, cDNA synthesis, and quantitative real-time polymerase chain reaction

RNA was isolated from MACS-sorted splenic B cells with the RNA Mini Kit (Qiagen, Hilden, Germany). Quantification of RNA was performed with a NanoPhotometer (Implen, Munich, Germany). The RNA was reverse transcribed to cDNA with the Quantitect Reverse Transcription kit (Qiagen). Gene expression analysis was performed with primer assays from Qiagen [*IL-10*] and Eurofins [*IL-12α*, *Ebi3*; *Tgfb2*, *B2m*; Table 1]. For quantitative analysis, the expression levels of all target genes were normalized against beta-2-microglobulin (*B2m*; Δ cycle threshold [Ct]). For *IL-10*, *IL-12α*, *Ebi3*, and *Tgfb2*, gene expression values were then calculated by the delta delta Ct (ΔΔCt) method, with the mean of the control group being the calibrator to which all other samples were compared. Relative quantities (RQs) were determined with the equation RQ = 2^−ΔΔCt^.

**Table 1:** Primers used for quantified reverse transcription PCR.

Gene	Forward primer	Reverse primer
*IL-12α*	5′-ATCACACGGGACCAAACCAG-3′	5′-CTGAAGTGCTGCGTTGAT-3′
*Ebi3*	5′-GTCAAGCTCAGGACCTCA-3′	5′-ACCCTGCCACCCTCAAGTAG-3′
*Tgfb2*	5′-TCACCACAAAGACAGGAACC-3′	5′-TACCTGCAAATCTCGCCTCG-3′
*B2m*	5′-GGCTCACACTGAATTCACCC-3′	5′-GTCTCGATCCCAGTAGACGG-3′

*B2m*, Beta-2 microglobulin; *Ebi3*, Epstein–Barr virus–induced gene 3; *IL*, interleukin, PCR, polymerase chain reaction; *Tgfb*, tumor growth factor beta.

### Immunofluorescence histology

For the visualization of catecholamines, B cells were treated with anti-IgM/CpG for 4 h and 24 h or left untreated. Afterward, cells were fixed in 2% PBS/formaldehyde before staining with NS_521 (10 μM, green) for 30 min at 37°C. The nucleus was stained with DAPI (blue), which was supplemented in ProLong Gold reagent (Invitrogen, Carlsbad, Germany).

### Inhibition of TH

TH activity was inhibited by treating B cells with different concentrations of 3-Iodo-L-tyrosine (Sigma-Aldrich, catalog number: 18250), 30 min before activation with anti-IgM/CpG.

### Flow cytometry

Splenic B cells were fixed with 2% PBS/formaldehyde for 20 min at room temperature (RT) and treated with FcR blocking reagent (Miltenyi Biotec, catalog number: 130-092-575) for 10 min to inhibit unspecific Fc receptor bindings. For cell surface stainings, B cells were either incubated for 30 min with the primary antibodies anti-NET, anti-VMAT-1 and anti-VMAT-2 to detect catecholamine transporters or with anti-ADRA1A, anti-ADRA1B, anti-ADRA2B, and anti-ADRB2 to evaluate the expression of ADRs. In addition, PE-conjugated anti-CD19 antibody was added for 10 min. Afterward, B cells were incubated with the secondary antibody Alexa488-conjugated goat anti-rabbit (1:2,000) for 30 min at 4°C. For all other surface staining’s, unfixed B cells were used and only fixed when additional intracellular markers were stained.

For intracellular staining of catecholamine transporters and TH, B cells were additionally permeabilized with Inside Perm (Miltenyi Biotec) and stained with the unlabeled primary antibodies anti-NET, anti-VMAT-1, and anti-VMAT-2 or with the directly anti–TH-PE-or anti-TH-Alexa488-conjugated antibody. After 30 min, cells were either incubated with the secondary goat anti-rabbit Alexa488-labeled antibody (1:2,000, for catecholamine transporters) for 30 min or were directly analyzed (TH) with the MacsQuant Analyzer 10 (Miltenyi Biotec). For intracellular cytokine stainings, B cells were activated or nonactivated with anti-IgM/CpG in the presence of 5 mg/mL brefeldin A (BFA, Sigma, catalog number: B6542) to prevent cytokine release. After 4 h, B cells were incubated with anti-CD19-VioBright FITC antibody for 10 min at 4°C, fixed with 2% PBS/formaldehyde for 20 min at RT and permeabilized with InsidePerm (Miltenyi Biotec) at 4°C followed by incubation with anti-IL-10-PE-conjugated antibody (Miltenyi Biotec).

For the detection of activation markers, unfixed B cells were incubated with anti-CD86-APC-Vio770, anti-MHC-II-PE and anti-CD40-APC in combination with anti-CD19-VioBright FITC for 10 min.

For all primary antibodies, relevant isotype control antibodies were used. Stained B cells were investigated with the MACSQuant Analyzer 10 (Miltenyi Biotec) and analyzed by using FlowJo and Flowlogic software.

### ELISA

Enzyme-linked immunosorbent assay (Mouse IL-10 ELISA Set; BD OptEIA, San Diego, California, USA, catalog number: 555282), 2-CAT (N-D) Research ELISA (ImmuSmol, Talence, France, catalog number: BA-E-5500) and Adrenaline/Epinephrine ELISA (LSBio, Seattle, Washington, USA, catalog number: LS-F5372) were conducted according to manufacturer’s instructions.

### T cell suppression assay

Splenocytes from immunized DBA/1J mice were labeled with the cell proliferation dye eFluor 450 (10 μM; eBioscience, Thermo Fisher Scientific, catalog number: 65-0842-90) and activated with 1 μg/mL soluble anti-CD3e (eBioscience, Thermo Fisher Scientific, Clone: eBio500A2) and 1 μg/mL soluble anti-CD28 (eBioscience, Thermo Fisher Scientific, Clone: 37.51) antibody. Autologous B cells were preactivated with CpG (1.25 μg/mL) for 4 h or left nonactivated in the presence or absence of ß-ADR agonists NE (10 μM) or isoproterenol (10 μM). After preactivation, B cells were washed twice with PBS before using them for coculture and transwell experiments. Activated and eFluor450-labeled splenocytes were cocultured in a 96 well plate (Greiner Bio-One, Solingen, Germany) with or without preactivated B cells at ratio of 1:2 in cRPMI medium, for 72 h at 37°C and 5% CO_2_.

For testing the influence of IL-10 and the expression of PD-L1 and FasL in the suppression of CD4+ T cells, B cells were either cocultured with splenocytes or separated by them by performing transwell experiments. For transwell experiments, preactivated B cells were placed into the upper chamber of a 96-well insert and the activated, eFluor450-labeled splenocytes were added into the lower chamber, separated with a membrane (pore size: 3 μm, Sigma-Aldrich) and both containing cRPMI medium. To evaluate the direct effect of IL-10 on proliferation of CD4+ T cells an anti-IL-10 depletion antibody (5 μg/mL) and rat IgG kappa (5 μg/mL) isotype control antibody was used in coculture and transwell experiments. After 72 h, cells were incubated with anti-CD19-VioBright-Fitc, anti-PD-L1-PE/Cy7, and anti-FasL-APC to analyze surface markers on B cells involved in T cell suppression and with CD4-APC-Vio770 to investigate T cell proliferation by flow cytometry (MACSQuant Analyzer 10, Miltenyi Biotec). Before analysis, propidium iodide (PI, Miltenyi Biotec) was added to the cells. The suppression of CD4+ T cell proliferation was determined by relative quantification of the mean fluorescence intensity (MFI) to the control group. Higher MFI in comparison to the control group indicate a suppression of CD4 T cell proliferation while a lower MFI shows an increase in CD4 T cell proliferation.

### Pulsing of B lymphocytes with CII

Splenic B cells and B cell–depleted splenocytes from mice with CIA were isolated by MACS. Splenocytes were labeled with the cell proliferation dye eFluor 450 (10 μM) and activated with soluble anti-CD3e (1 μg/ml) and soluble anti-CD28 (1μg/ml) antibody. Autologous B cells were cultured in the presence of CII antigen or peptide (50 μg/ml and 100 μg/ml). After 24 h, CII-pulsed B cells were washed twice with PBS and were cocultured with the activated, B cell–depleted splenocytes for 72 h at 37°C and 5% CO_2_ at ratio of 1:2 in a 96-well plate. The CD4+ T cell proliferation was monitored by flow cytometry. CII antigen was incubated for 30 min at 56°C to obtain CII peptide.

### Annexin V-FITC binding assay

Untreated (control) and 3-Iodo-L-tyrosine-treated B cells were labeled by using the Annexin V Fitc Apoptosis detection kit (BD, Heidelberg, Germany) according to manufacturer’s instructions. Before analysis PI (Miltenyi Biotec) was added to B cells and then analyzed by flow cytometry (MacsQuant Analyzer 10; Miltenyi Biotec). The double labeling procedure allows a distinction into living (Annexin V -/PI-), early apoptotic (Annexin V +/PI-), late apoptotic (Annexin V+/PI+), and necrotic cells (Annexin V -/PI+).

### Western blot analysis

Isolated splenic B cells from naive DBA1/J mice were homogenized in lysis buffer (50 mM Tris-HCl, pH 8.0; 150 mM NaCl; 2 mM EDTA; 1% NP-40; 0,1% SDS; 1% sodiumdeoxycholate; 1 mM sodiumorthovanadate; 1 mM PMSF; 50 mM sodiumfluoride; protease-inhibitor-cocktail (1x)) for 5 min on ice, snap-frozen in liquid nitrogen (freeze/thaw cycle 2x) and centrifuged for 10 min by 10,000 g at 4°C. Protein concentration was determined in the cell supernatant, mixed with Rotiload (1:4, Bio-Rad, Feldkirchen, Germany) and boiled for 5 min at 95°C. Total B cell protein (10 μg) was separated by 12.5% SDS polyacrylamide gel electrophoresis (SDS-PAGE) and blotted at 30 mA for 90 min on nitrocellulose membranes (Bio-Rad). After blocking with 5% (w/v) blotting-grade nonfat dry milk (Bio-Rad) for 1 h at RT, membrane was washed with TBST and incubated overnight at 4°C followed by incubation with primary antibody: anti-TH (1:1,000, Merck Millipore, AB152). After washing with TBST, membrane was incubated with horseradish peroxidase (HRP)-conjugated secondary antibody (1:2,000, Dako, Hamburg, Germany, catalog number: P0448) for 2h at RT. As reference, glycerinaldehyd-3-phosphat-dehydrogenase (GAPDH) was used to calculate the levels of target proteins. For the visualization, the proteins ECL-solution (Luminol, Para-Hydroxycoumarinacid, DMSO) and ChemiDoc Touch Imaging System (Bio-Rad) was used. Protein spots were analyzed with the Image Lab 5.2.1 software (Bio-Rad).

### Statistics

Statistical analysis was performed using Prism 8 (GraphPad Software, La Jolla, California, USA). Differences among 2 groups of samples were tested for statistical significance by applying 2-tailed Student *t* test. Comparisons involving more than 2 groups of samples, statistical significance was evaluated by 1-way analysis of variance (ANOVA). Student *t* test or 1-way ANOVA with Welch correction was performed if variance was significantly different between 2 sets of data. For each experiment, the specific statistical test is mentioned in the figure legend. If not otherwise stated, each data point indicates B cells from individual mice. The sample size (*n*) is indicated in the figure legends. Data are pooled from at least 2 independent experiments unless mentioned otherwise in the figure legend. Data are presented as mean ± SD. The level of statistical significance was set at n.s., not significant; *p < 0.05, **p < 0.01, ***p < 0.001, or ****p < 0.0001.

### Ethics statement

Animal experiments were carried out with approval of the “Landesamt für Natur, Umwelt und Verbraucherschutz Nordrhein-Westfalen” (LANUV), Germany (approval number: 84–02.04.2016.A281 and 84–02.04.2016.A283) in accordance with the German laws for animal protection. Animal care and documentation was supervised by the “Zentrale Einrichtung für Tierforschung und wissenschaftliche Tierschutzaufgaben (ZETT)” of the Heinrich-Heine-University Düsseldorf, Düsseldorf, Germany.

## Supporting information

S1 Data This file contains data underlying figures and Supporting information figures. (XLSX)

S1 Fig Activation with anti-IgM/CpG increase TH and PNMT expression in B cells. (**A**, **B**) B cells were activated with anti-IgM/CpG for 24 or 48 h or left nonactivated (0 h). The expression of TH (A) and PNMT (B) was analyzed by western blot. One of 3 representative full western blot images with TH (A), PNMT (B) and GAPDH (A, B) expression is shown (*n* = 3). For underlying data, see S1 Data. GAPDH, glycerinaldehyd-3-phosphat-dehydrogenase; PNMT, phenylethanolamine N-methyltransferase; TH, tyrosine hydroxylase.
(PDF)

S2 Fig B cells from C57BL/6 and BALB/c mice raise TH and IL-10 expression after activation. (**A**, **B**) Splenic B cells from C57BL/6 and BALB/c mice were activated with anti-IgM/CpG for 24 h or 48 h or left nonactivated (0 h). The expression of TH (A) and IL-10 (B) was measured by flow cytometry (*n* = 4). The gating strategy and one representative dot plot for each time point is shown. Ordinary 1-way ANOVA followed by Tukey multiple comparisons test (BALB/c) and Brown–Forsythe and Welch ANOVA followed by Tamhane T2 multiple comparison test (C57BL/6) was used for comparisons. *p < 0.5; **p < 0.01; ***p < 0.001; ****p < 0.0001. For underlying data, see S1 Data. ANOVA, analysis of variance; TH, tyrosine hydroxylase.
(PDF)

S3 Fig Higher concentrations of TD-/TI-stimuli increase B cell activation. (**A**, **B**) B cells were activated with different concentrations of the TD mitogen (A: anti-CD40/IL-4) or TI mitogens (B: Poly I:C, TLR3; LPS, TLR4) for 24 h. As control group, nonactivated B cells were used. The MFI of B cell activation markers MHC-II, CD86 and CD40 were determined on the surface of B cells by flow cytometry (A; *n* = 6 and B; *n* = 4). For the experiments B cells from naive DBA/1J mice were used. Ordinary 1-way ANOVA was used for comparisons. n.s., not significant; *p < 0.5; **p < 0.01; ***p < 0.001; ****p < 0.0001. For underlying data, see S1 Data. ANOVA, analysis of variance; LPS, lipopolysaccharide; MFI, median fluorescence intensity; MHC-II, major histocompatibility complex-II; Poly I:C, polyinosinic:polycytidylic acid; TD, T cell–dependent; TI, T cell–independent; TLR, Toll-like receptor.
(PDF)

S4 Fig Higher concentrations of TD-/TI-stimuli raise TH and IL-10 expression. (**A**, **B**) B cells were activated with different concentrations of the TD mitogen anti-CD40/IL-4 or different concentrations of the TI mitogens TLR3 (Poly I:C) or TLR4 (LPS) for 24 h. As control group, nonactivated B cells were used. (**A**) The expression of CD19+TH+ B cells was measured by flow cytometry (TD: *n* = 6; TI: *n* = 4) and (**B**) the production of IL-10 was analyzed by ELISA (TD: *n* = 6; TI: LPS: *n* = 6; Poly I:C: *n* = 4). B cells from naive DBA/1J mice were used for the experiments. Statistical significance was determined by Brown–Forsythe and Welch ANOVA tests followed by Dunnett T3 multiple comparison (A, B: IL-4, anti-CD40 and IL-4/anti-CD40) or ordinary 1-way ANOVA followed by Dunnett multiple comparison test (A, B: LPS and Poly I:C). n.s., not significant; *p < 0.5; **p < 0.01; ****p < 0.0001. For underlying data, see S1 Data. ELISA, enzyme-linked immunosorbent assay; LPS, lipopolysaccharide; Poly I:C, polyinosinic:polycytidylic acid; TD, T cell–dependent; TH, tyrosine hydroxylase; TI, T cell–independent; TLR, Toll-like receptor.
(PDF)

S5 Fig CpG-ODN 1826 increases activation markers, TH and IL-10 expression. (**A**–**C**) B cells were activated with different classes of CpG-ODNs: ODN 1585 (class A); ODN 1826 (class B) and ODN 2395 (class C) for 24 h. Nonactivated B cells treated with C-ODNs were used as controls. The MFI of B cell activation markers MHC-II, CD86 and CD40 (A; *n* = 4) and the expression of CD19+TH+ B cells (B; *n* = 4) were determined by flow cytometry. The amount of IL-10 in cell culture supernatants was determined by ELISA (C; *n* = 8). For the experiments B cells from naive DBA/1J mice were used. Data are pooled from 4 experiments (C). Student *t* test (A-C) was used for comparisons. n.s., not significant; *p < 0.5; **p < 0.01; ***p < 0.001; ****p < 0.0001. For underlying data, see S1 Data. C-ODNs, control oligodeoxynucleotides; ELISA, enzyme-linked immunosorbent assay; MFI, median fluorescence intensity; MHC-II, major histocompatibility complex-II; ODNs, oligodeoxynucleotides; TH, tyrosine hydroxylase.
(PDF)

S6 Fig B cells express ADRs and transporters. (**A**, **B**) B cells were activated with anti-IgM/CpG for 24 h and 48 h. Nonactivated B cells were used as control group (0 h). The gating strategy for the detection of ADRA1B (A) and ADRs and TH expression, including representative dot plots of all investigated ADRs (B) are shown. (**C**) Nonactivated B cells were used for the detection of monoamine transporters. The gating strategy for the detection of VMAT-1 is shown. ADRA1B, adrenergic receptor alpha 1b; TH, tyrosine hydroxylase; VMAT-1, vesicular monoamine transporter 1.
(PDF)

S7 Fig B cells from DBA/1J mice increase IL-10 expression after activation. (**A**, **B**) 2.5 × 10^5^ splenic B cells from DBA/1J mice were activated with anti-IgM/CpG for 24 h and 48 h or were left nonactivated (0 h). The expression of IL-10 was measured by flow cytometry (A; *n* = 4) and in the supernatant of cultured B cells (B; *n* = 4). One representative dot plot for each time point is shown (A). Statistical significance was determined by ordinary 1-way ANOVA followed by Tukey multiple comparison test (A, B). *p < 0.5; ****p < 0.0001. For underlying data, see S1 Data. ANOVA, analysis of variance; IL, interleukin.
(PDF)

S8 Fig Bregs and MZ B cells expand after anti-IgM/CpG activation. (**A**, **B**) B cells were activated with anti-IgM/CpG or left untreated. After 48 h, Bregs (CD19+CD1d+, Tim-1+, CD5+ and CD5-) (A) and MZ B cells (B220+, CD1d+, CD21/35+/CD9+ and CD9-) (B) were analyzed for TH and IL-10 expression by flow cytometry and compared to control B cells (0 h). The gating strategy is shown. Breg, regulatory B cell; MZ, marginal zone; TH, tyrosine hydroxylase.
(PDF)

S9 Fig T2-MZP cells and plasmablasts expand after anti-IgM/CpG activation. (**A**, **B**) B cells were activated with anti-IgM/CpG or left untreated. After 48 h, T2-MZP (CD19+, CD1d^hi^, CD24^hi^, CD23^hi^ and CD23-) (A) and plasmablasts (B220+CD44+CD138+ and CD138-) (B) were analyzed for TH and IL-10 expression by flow cytometry and compared to control B cells (0 h). The gating strategy is shown.TH, tyrosine hydroxylase.
(PDF)

S10 Fig TH inhibitor has no effect on B cell survival. (**A**, **B**) B cells were treated for 30 min with 0.5 mM of the TH inhibitor 3-Iodo-L-tyrosine before activation with anti-IgM/CpG for 24 h. As control group B cells were left untreated. (**A**) The expression of living (PI-Annexin V-), early apoptotic (PI-Annexin V+), late apoptotic (PI^+^Annexin V^+^) and necrotic cells (PI+Annexin V-) was analyzed with the Annexin V-FITC binding assay by flow cytometry (*n* = 6). One of 6 representative dot plots is shown. (**B**) The frequency of living B cells was determined by flow cytometry (*n* = 6). For the experiments B cells from naive DBA/1J mice were used. Student *t* test (B) was used for comparisons. n.s., not significant. For underlying data, see S1 Data. PI, propidium iodide; TH, tyrosine hydroxylase.
(PDF)

S11 Fig CII antigen-primed B cells suppress CD4 T cell proliferation. Splenic B cells and B cell–depleted splenocytes from CIA mice were isolated by MACS. Splenocytes were labeled with the cell proliferation dye eFluor 450 (10 μM) and activated with soluble anti-CD3e (1 μg/ml) and soluble anti-CD28 (1 μg/ml) antibody. Autologous B cells were cultured in the presence of CII antigen or peptide (50 μg/ml and 100 μg/ml) for 24 h before cocultured with activated, B cell–depleted splenocytes for 72 h at 37°C and 5% CO_2_ at ratio of 1:2 (B cells:Splenocytes) in a 96 well plate. CD4+ T cell proliferation was monitored by flow cytometry (*n* = 6). One representative histogram is shown. Ordinary 1-way ANOVA was used for comparisons. n.s., not significant; *p < 0.5; **p < 0.01; ****p < 0.0001. For underlying data, see S1 Data. Ag, antigen; ANOVA, analysis of variance; CII, type II collagen; CIA, collagen-induced arthritis; MACS, magnetic-activated cell sorting; Pep, peptide.
(PDF)

S12 Fig ß-ADR–derived IL-10 increases the suppression of CD4 T cell proliferation. B cells from WT and *IL-10*^−/−^ mice were isolated by MACS. Splenocytes were labeled with the cell proliferation dye eFluor 450 (10 μM) and activated with soluble anti-CD3e (1 μg/ml) and soluble anti-CD28 (1 μg/ml) antibody. B cells were activated with CpG or additionally treated with NE or Isopr. for 4 h, before cocultured with activated autologous splenocytes. Nonactivated and nontreated B cells were used as control group. CD4+ T cell proliferation was monitored by flow cytometry (*n* = 5). The PI has been calculated by using the number of events measured in each division peak. Ordinary 1-way ANOVA was used for comparisons. n.s., not significant; **p < 0.01; ***p < 0.001; ****p < 0.0001. For underlying data, see S1 Data. ANOVA, analysis of variance; Isopr., isoproterenol MACS, magnetic-activated cell sorting; NE, norepinephrine; PI, proliferation index.
(PDF)

## Abbreviations

ADR: adrenergic receptor
ANOVA: analysis of variance
APC: antigen-presenting cell
BCR: B cell receptor
Breg: regulatory B cell
CFA: complete Freund’s adjuvant
CIA: collagen-induced arthritis
CII: type II collagen
EAE: experimental autoimmune encephalomyelitis
FasL: Fas ligand
FBS: fetal bovine serum
GAPDH: glycerinaldehyd-3-phosphat-dehydrogenase
HRP: horseradish peroxidase
IL: interleukin
Isopr: Isoproterenol
L-Dopa: L-3,4-dihydroxyphenylalanine
LPS: lipopolysaccharide
MACS: magnetic-activated cell sorting
MFI: mean or median fluorescence intensity
MHC-II: major histocompatibility complex class II
NE: norepinephrine
NET: norepinephrine transporter
NS_521: neurosensor_521
ODN: oligodeoxynucleotide
PD-L1: programmed death ligand 1
PI: propidium iodide
PNMT: phenylethanolamine N-methyltransferase
Poly I:C: polyinosinic-polycytidylic acid
qRT-PCR: quantitative real-time polymerase chain reaction
RA: rheumatoid arthritis
RQ: relative quantity
SNS: sympathetic nervous system
TD: T cell–dependent
TGF-ß: tumor growth factor beta
TH: tyrosine hydroxylase
TI: T cell–independent
TLR: Toll-like receptor
VMAT: vesicular monoamine transporter
WT: wild-type
