# Supplementary figures and images for: Endogenously produced catecholamines improve the regulatory function of TLR9-activated B cells
Source: PLoS Biol. 2022 Jan 24;20(1):e3001513. doi: 10.1371/journal.pbio.3001513 (PMC8786184; doi:10.1371/journal.pbio.3001513)

# S1 Figure

**A**

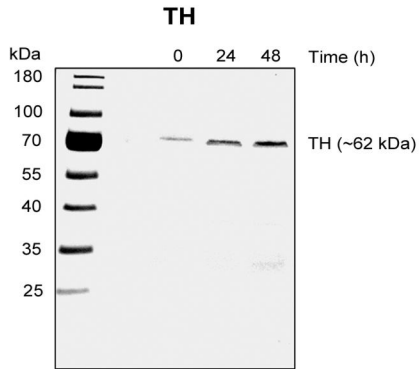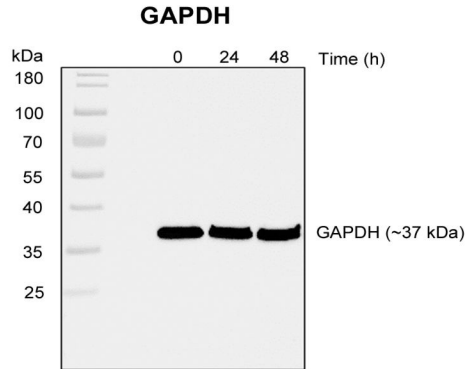

**B**

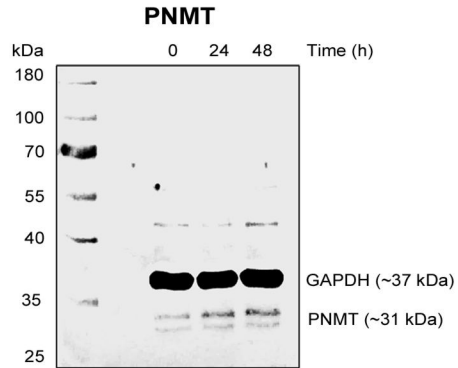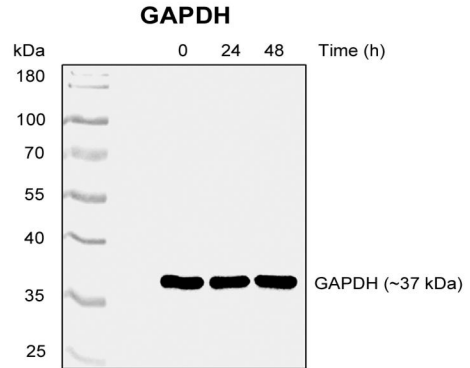

Supplement: S1 Fig — (A, B) B cells were activated with anti-IgM/CpG for 24 or 48 h or left nonactivated (0 h). The expression of TH (A) and PNMT (B) was analyzed by western blot. One of 3 representative full western blot images with TH (A), PNMT (B) and GAPDH (A, B) expression is shown (n = 3). For underlying data, see S1 Data. GAPDH, glycerinaldehyd-3-phosphat-dehydrogenase; PNMT, phenylethanolamine N-methyltransferase; TH, tyrosine hydroxylase. (PDF) [file pbio.3001513.s002.pdf]

# S2 Figure

**A**

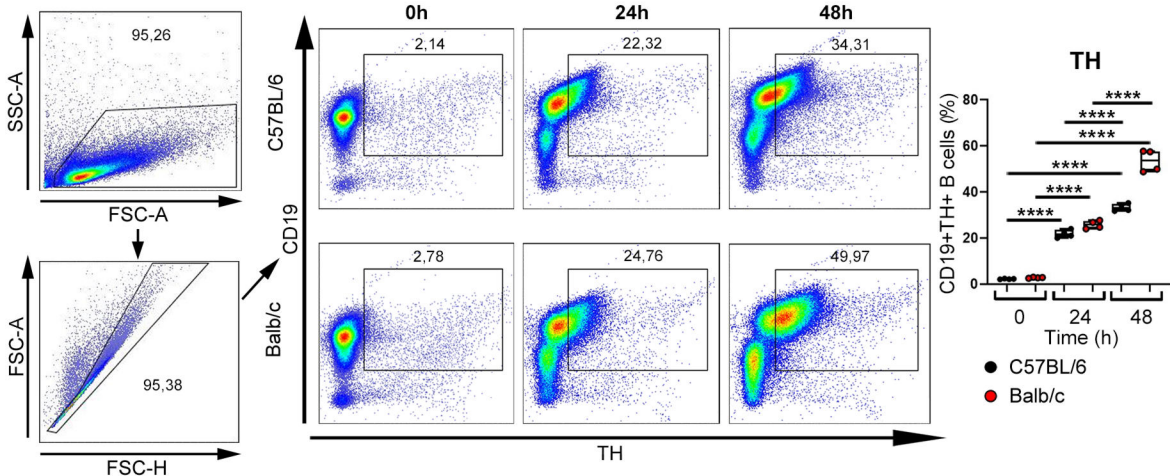

**B**

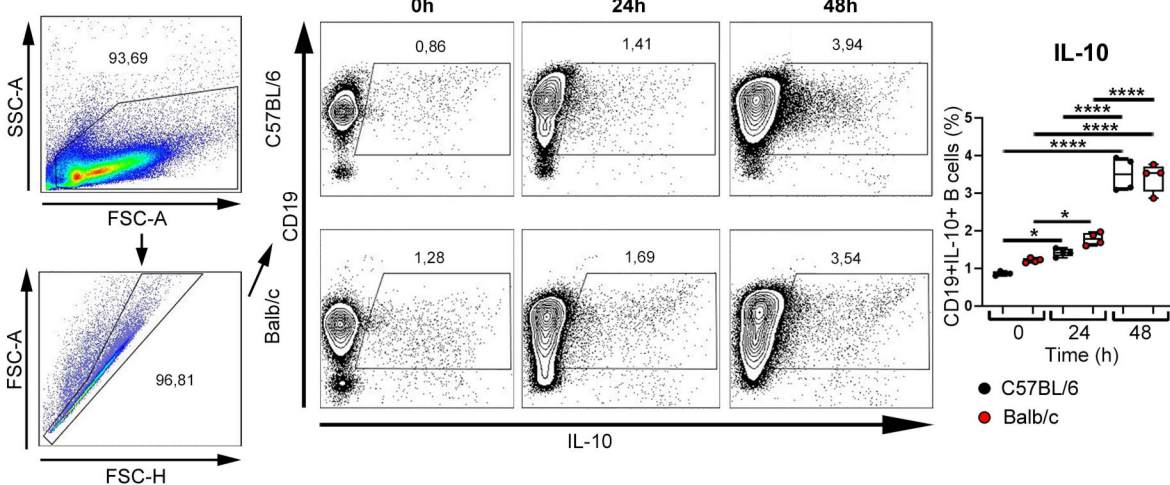

Supplement: S2 Fig — (A, B) Splenic B cells from C57BL/6 and BALB/c mice were activated with anti-IgM/CpG for 24 h or 48 h or left nonactivated (0 h). The expression of TH (A) and IL-10 (B) was measured by flow cytometry (n = 4). The gating strategy and one representative dot plot for each time point is shown. Ordinary 1-way ANOVA followed by Tukey multiple comparisons test (BALB/c) and Brown–Forsythe and Welch ANOVA followed by Tamhane T2 multiple comparison test (C57BL/6) was used for comparisons. *p < 0.5; **p < 0.01; ***p < 0.001; ****p < 0.0001. For underlying data, see S1 Data. ANOVA, analysis of variance; TH, tyrosine hydroxylase. (PDF) [file pbio.3001513.s003.pdf]

# S3 Figure

**A**

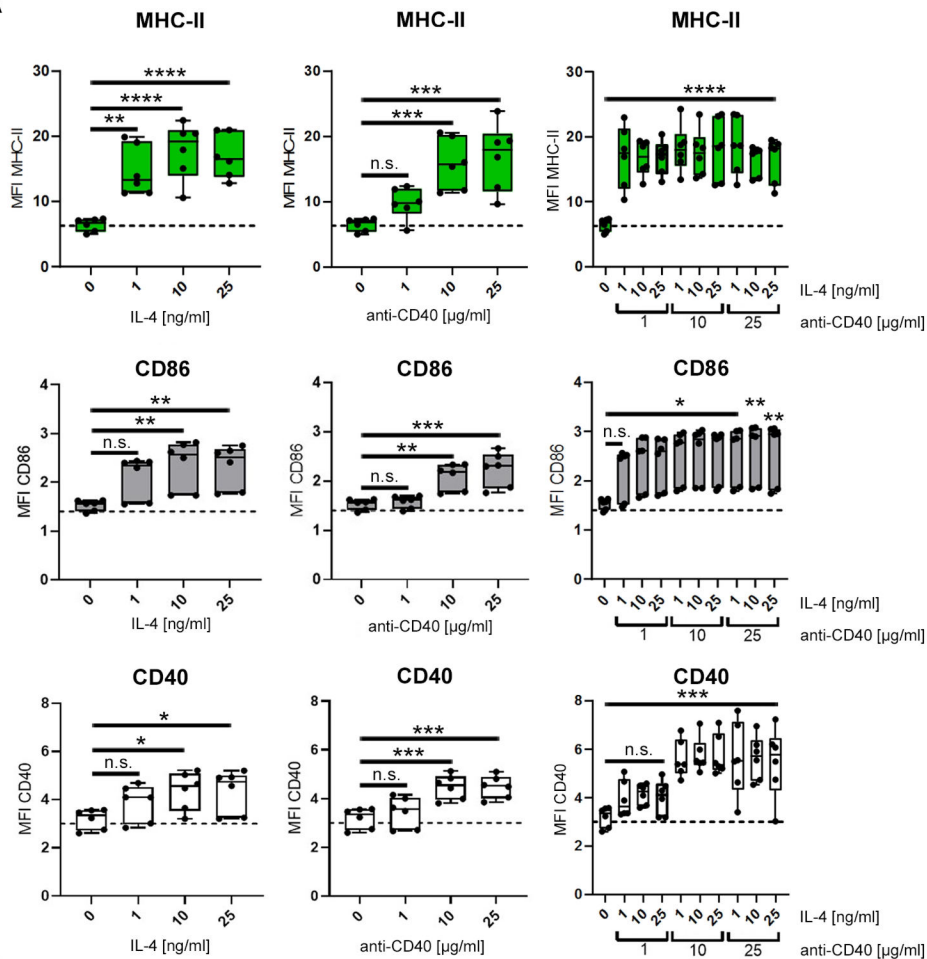

**B**

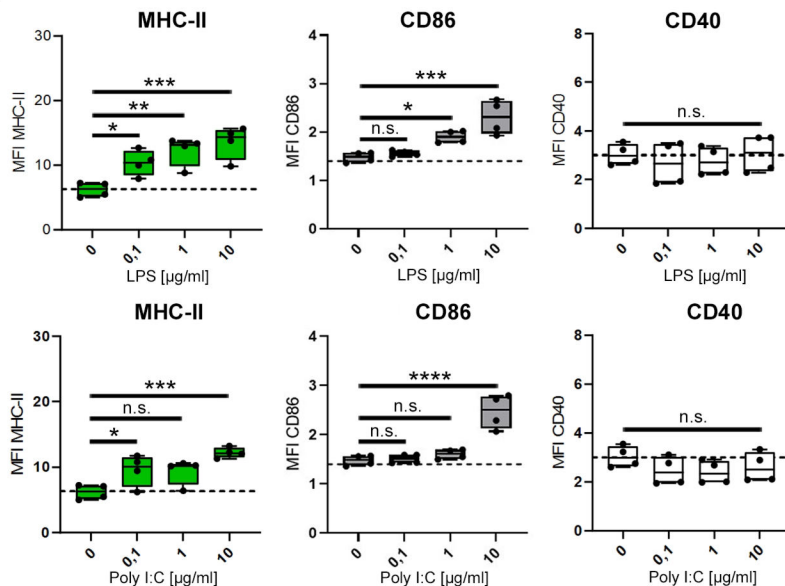

Supplement: S3 Fig — (A, B) B cells were activated with different concentrations of the TD mitogen (A: anti-CD40/IL-4) or TI mitogens (B: Poly I:C, TLR3; LPS, TLR4) for 24 h. As control group, nonactivated B cells were used. The MFI of B cell activation markers MHC-II, CD86 and CD40 were determined on the surface of B cells by flow cytometry (A; n = 6 and B; n = 4). For the experiments B cells from naive DBA/1J mice were used. Ordinary 1-way ANOVA was used for comparisons. n.s., not significant; *p < 0.5; **p < 0.01; ***p < 0.001; ****p < 0.0001. For underlying data, see S1 Data. ANOVA, analysis of variance; LPS, lipopolysaccharide; MFI, median fluorescence intensity; MHC-II, major histocompatibility complex-II; Poly I:C, polyinosinic:polycytidylic acid; TD, T cell–dependent; TI, T cell–independent; TLR, Toll-like receptor. (PDF) [file pbio.3001513.s004.pdf]

S4 Figure

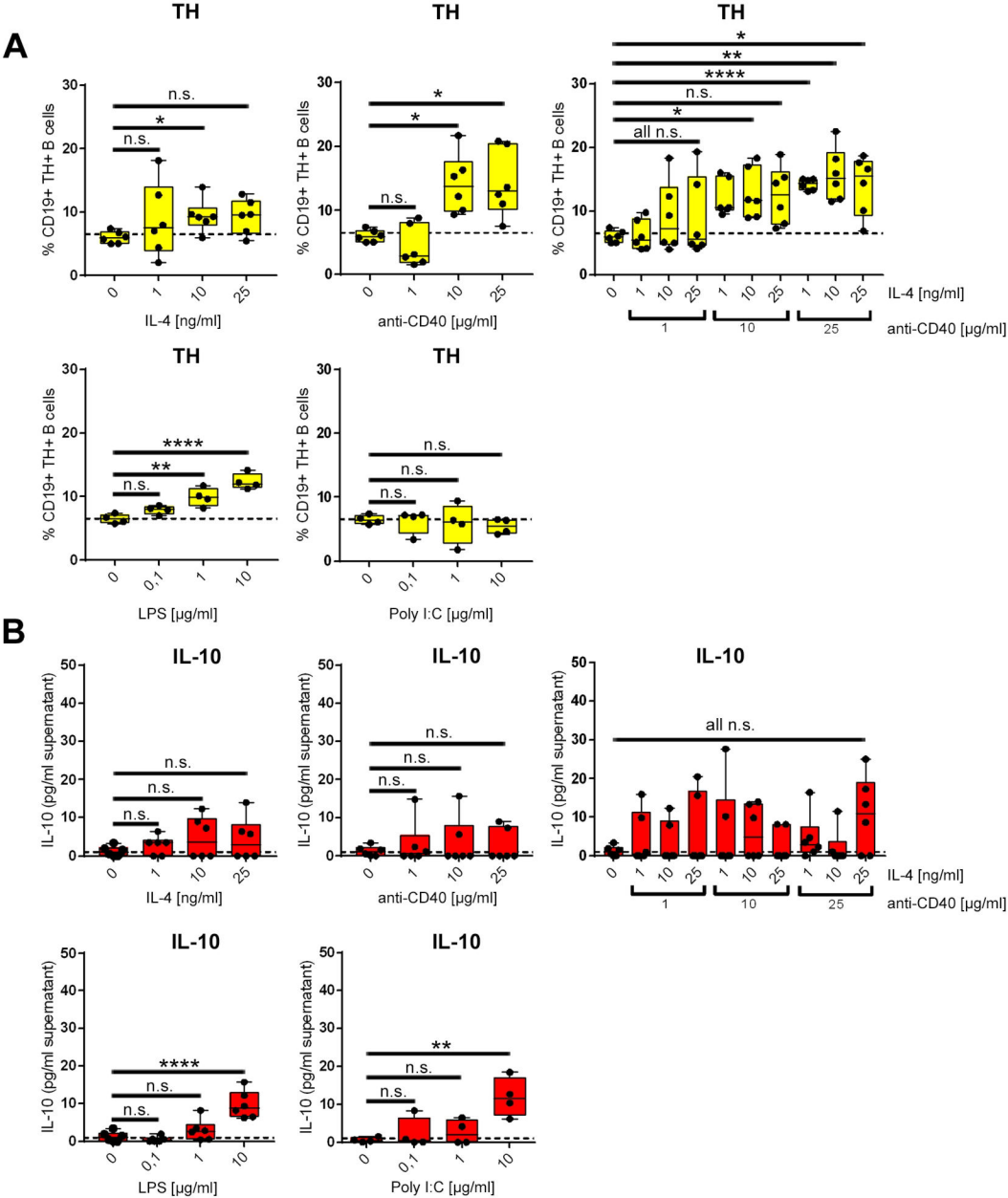

Supplement: S4 Fig — (A, B) B cells were activated with different concentrations of the TD mitogen anti-CD40/IL-4 or different concentrations of the TI mitogens TLR3 (Poly I:C) or TLR4 (LPS) for 24 h. As control group, nonactivated B cells were used. (A) The expression of CD19+TH+ B cells was measured by flow cytometry (TD: n = 6; TI: n = 4) and (B) the production of IL-10 was analyzed by ELISA (TD: n = 6; TI: LPS: n = 6; Poly I:C: n = 4). B cells from naive DBA/1J mice were used for the experiments. Statistical significance was determined by Brown–Forsythe and Welch ANOVA tests followed by Dunnett T3 multiple comparison (A, B: IL-4, anti-CD40 and IL-4/anti-CD40) or ordinary 1-way ANOVA followed by Dunnett multiple comparison test (A, B: LPS and Poly I:C). n.s., not significant; *p < 0.5; **p < 0.01; ****p < 0.0001. For underlying data, see S1 Data. ELISA, enzyme-linked immunosorbent assay; LPS, lipopolysaccharide; Poly I:C, polyinosinic:polycytidylic acid; TD, T cell–dependent; TH, tyrosine hydroxylase; TI, T cell–independent; TLR, Toll-like receptor. (PDF) [file pbio.3001513.s005.pdf]

# S5 Figure

**A**

**MHC-II**

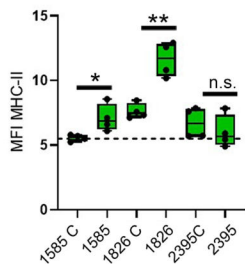

**CD86**

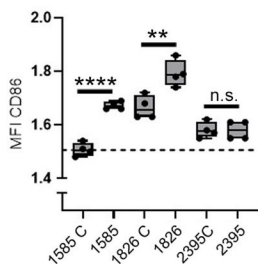

**CD40**

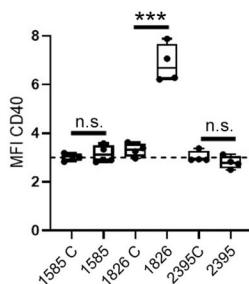

**B**

**TH**

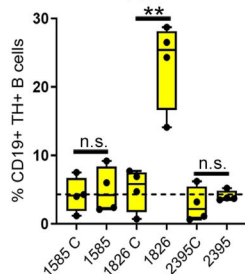

**C**

**IL-10**

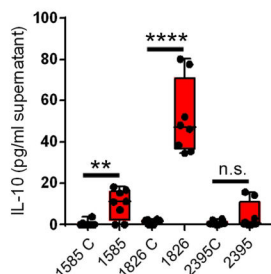

Supplement: S5 Fig — (A–C) B cells were activated with different classes of CpG-ODNs: ODN 1585 (class A); ODN 1826 (class B) and ODN 2395 (class C) for 24 h. Nonactivated B cells treated with C-ODNs were used as controls. The MFI of B cell activation markers MHC-II, CD86 and CD40 (A; n = 4) and the expression of CD19+TH+ B cells (B; n = 4) were determined by flow cytometry. The amount of IL-10 in cell culture supernatants was determined by ELISA (C; n = 8). For the experiments B cells from naive DBA/1J mice were used. Data are pooled from 4 experiments (C). Student t test (A-C) was used for comparisons. n.s., not significant; *p < 0.5; **p < 0.01; ***p < 0.001; ****p < 0.0001. For underlying data, see S1 Data. C-ODNs, control oligodeoxynucleotides; ELISA, enzyme-linked immunosorbent assay; MFI, median fluorescence intensity; MHC-II, major histocompatibility complex-II; ODNs, oligodeoxynucleotides; TH, tyrosine hydroxylase. (PDF) [file pbio.3001513.s006.pdf]

# S6 Figure

**A**

**Gating Strategy**

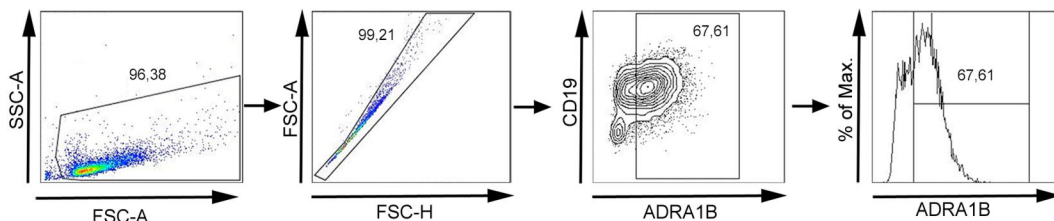

**B**

**Gating Strategy**

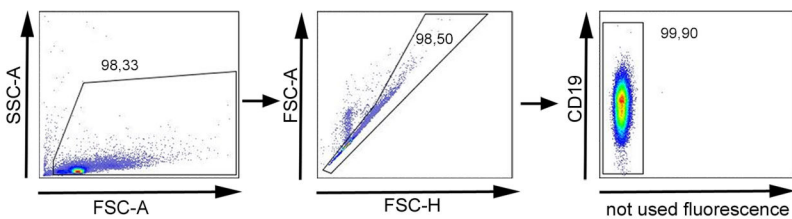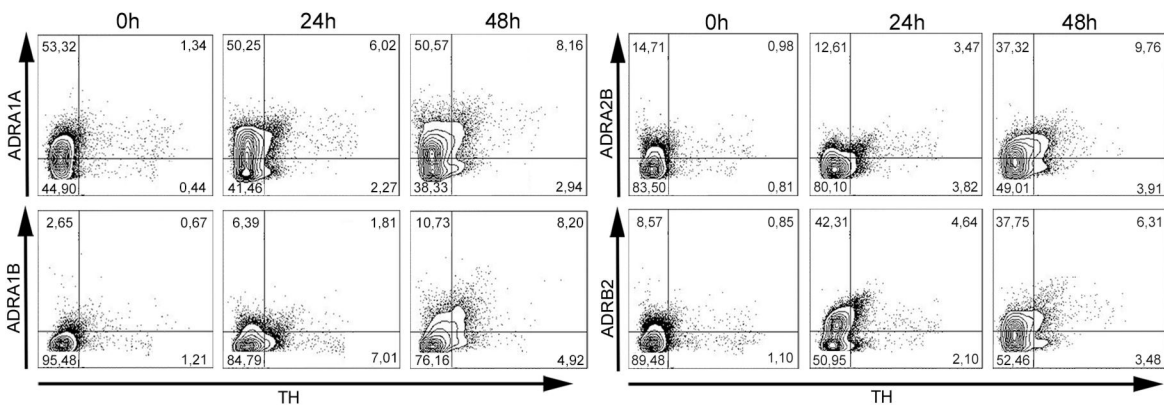

**C**

**Gating Strategy**

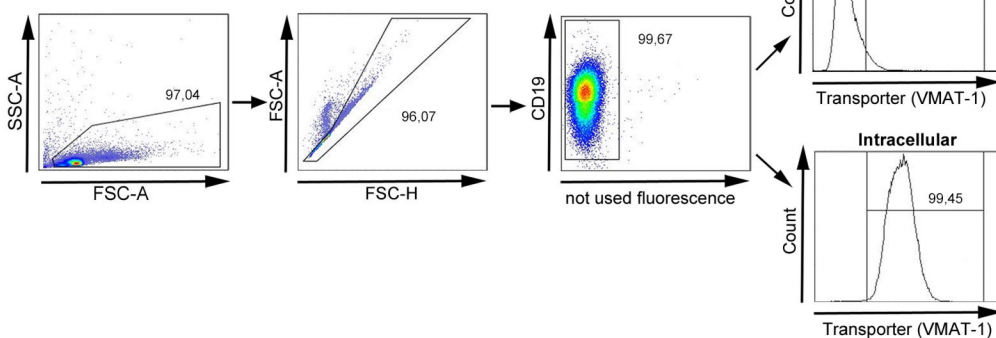

Supplement: S6 Fig — (A, B) B cells were activated with anti-IgM/CpG for 24 h and 48 h. Nonactivated B cells were used as control group (0 h). The gating strategy for the detection of ADRA1B (A) and ADRs and TH expression, including representative dot plots of all investigated ADRs (B) are shown. (C) Nonactivated B cells were used for the detection of monoamine transporters. The gating strategy for the detection of VMAT-1 is shown. ADRA1B, adrenergic receptor alpha 1b; TH, tyrosine hydroxylase; VMAT-1, vesicular monoamine transporter 1. (PDF) [file pbio.3001513.s007.pdf]

# S7 Figure

A

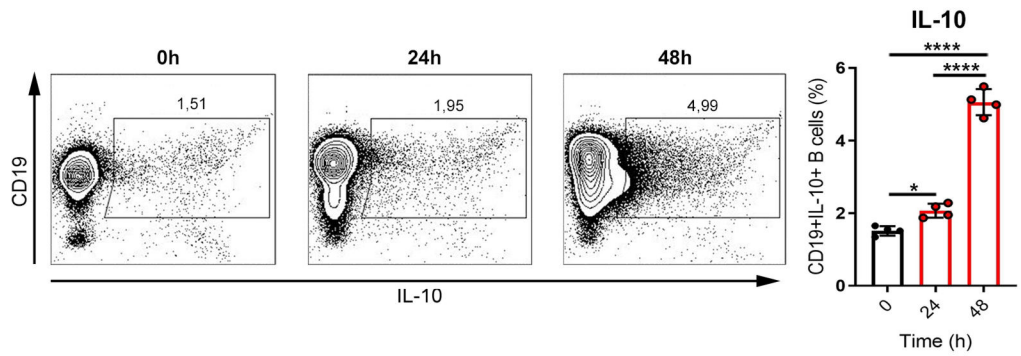

B

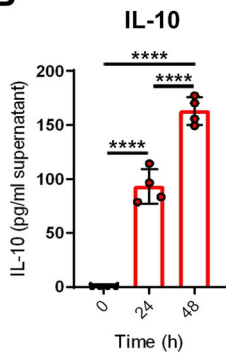

Supplement: S7 Fig — (A, B) 2.5 × 10^5^ splenic B cells from DBA/1J mice were activated with anti-IgM/CpG for 24 h and 48 h or were left nonactivated (0 h). The expression of IL-10 was measured by flow cytometry (A; n = 4) and in the supernatant of cultured B cells (B; n = 4). One representative dot plot for each time point is shown (A). Statistical significance was determined by ordinary 1-way ANOVA followed by Tukey multiple comparison test (A, B). *p < 0.5; ****p < 0.0001. For underlying data, see S1 Data. ANOVA, analysis of variance; IL, interleukin. (PDF) [file pbio.3001513.s008.pdf]

# S8 Figure

**A**

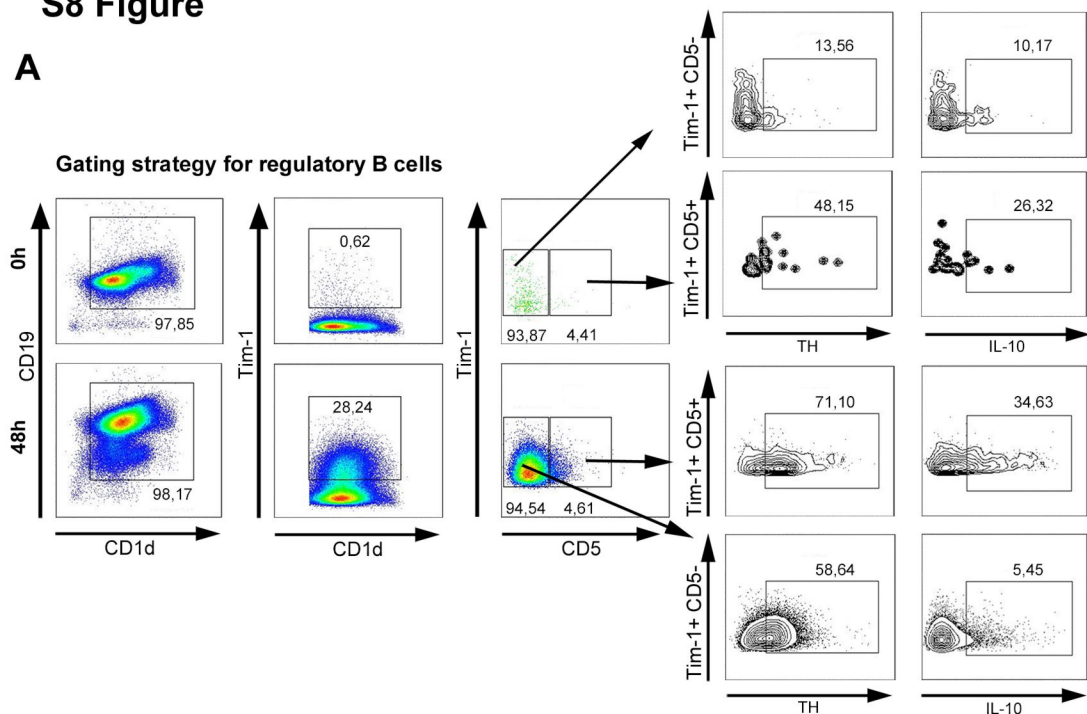

**B**

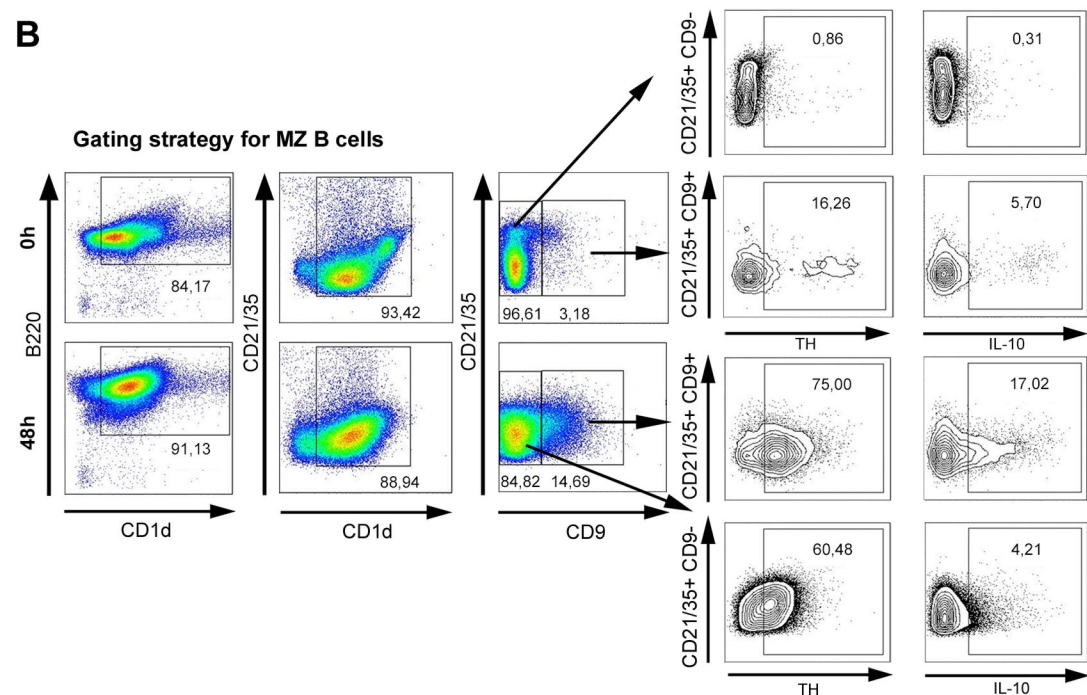

Supplement: S8 Fig — (A, B) B cells were activated with anti-IgM/CpG or left untreated. After 48 h, Bregs (CD19+CD1d+, Tim-1+, CD5+ and CD5-) (A) and MZ B cells (B220+, CD1d+, CD21/35+/CD9+ and CD9-) (B) were analyzed for TH and IL-10 expression by flow cytometry and compared to control B cells (0 h). The gating strategy is shown. Breg, regulatory B cell; MZ, marginal zone; TH, tyrosine hydroxylase. (PDF) [file pbio.3001513.s009.pdf]

# S9 Figure

**A**

Gating strategy T2-MZP B cells

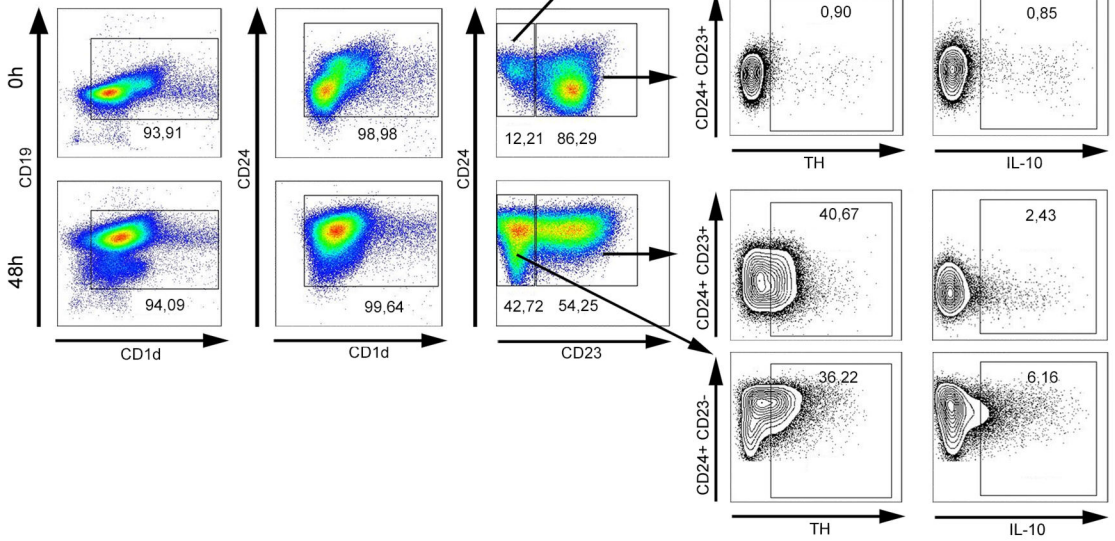

**B**

Gating strategy for plasmablasts

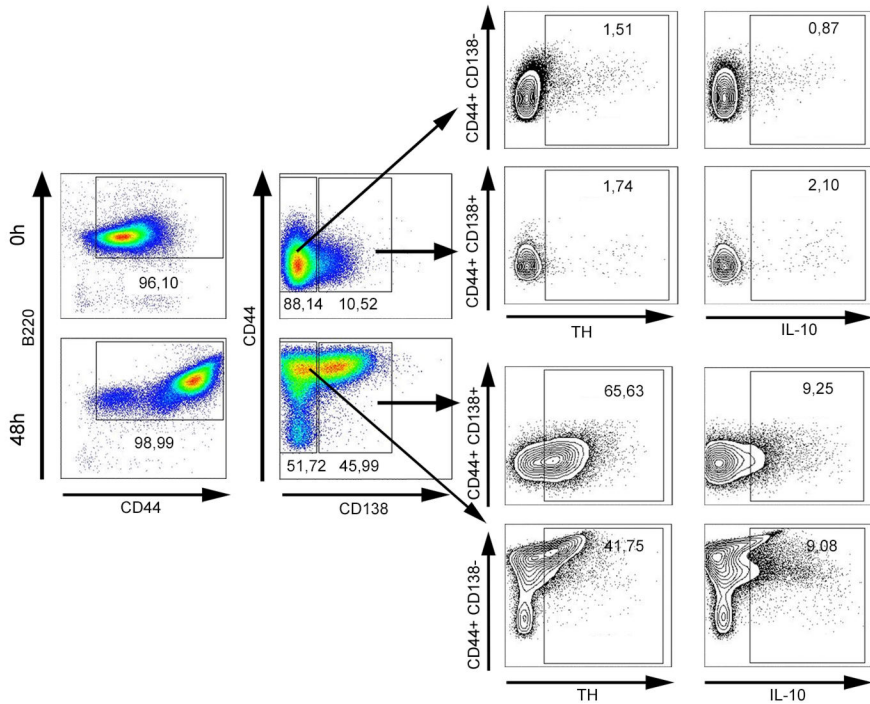

Supplement: S9 Fig — (A, B) B cells were activated with anti-IgM/CpG or left untreated. After 48 h, T2-MZP (CD19+, CD1dhi, CD24hi, CD23hi and CD23-) (A) and plasmablasts (B220+CD44+CD138+ and CD138-) (B) were analyzed for TH and IL-10 expression by flow cytometry and compared to control B cells (0 h). The gating strategy is shown.TH, tyrosine hydroxylase. (PDF) [file pbio.3001513.s010.pdf]

S10 Figure

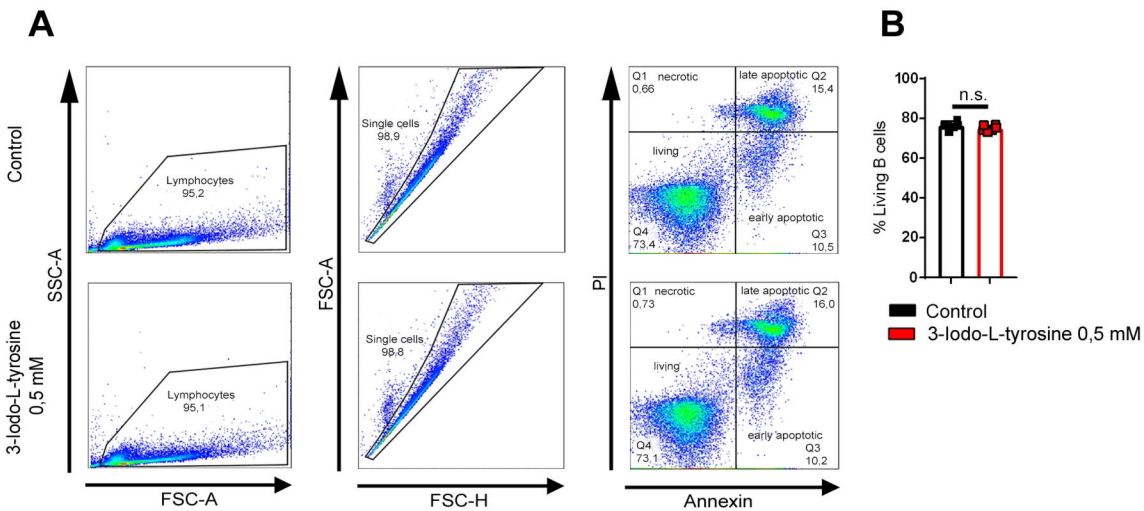

Supplement: S10 Fig — (A, B) B cells were treated for 30 min with 0.5 mM of the TH inhibitor 3-Iodo-L-tyrosine before activation with anti-IgM/CpG for 24 h. As control group B cells were left untreated. (A) The expression of living (PI-Annexin V-), early apoptotic (PI-Annexin V+), late apoptotic (PI+Annexin V+) and necrotic cells (PI+Annexin V-) was analyzed with the Annexin V-FITC binding assay by flow cytometry (n = 6). One of 6 representative dot plots is shown. (B) The frequency of living B cells was determined by flow cytometry (n = 6). For the experiments B cells from naive DBA/1J mice were used. Student t test (B) was used for comparisons. n.s., not significant. For underlying data, see S1 Data. PI, propidium iodide; TH, tyrosine hydroxylase. (PDF) [file pbio.3001513.s011.pdf]

# S12 Figure

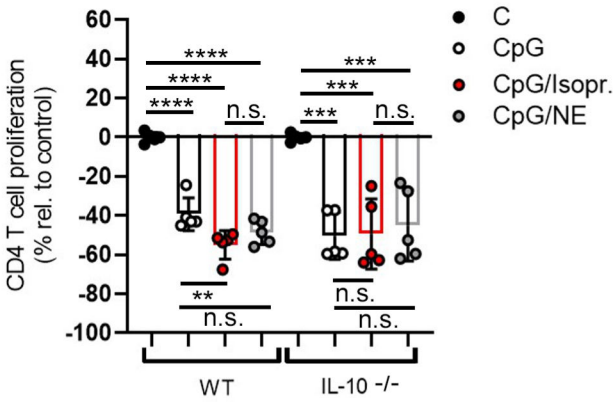

| Proliferation Index (PI) |               |                      |
|--------------------------|---------------|----------------------|
|                          | WT            | IL-10 <sup>-/-</sup> |
| C                        | 2.658 ± 0.267 | 2.778 ± 0.086        |
| CpG                      | 2.090 ± 0.232 | 2.146 ± 0.165        |
| CpG/Isopr.               | 2.008 ± 0.237 | 2.164 ± 0.207        |
| CpG/NE                   | 2.032 ± 0.239 | 2.184 ± 0.208        |

Supplement: S12 Fig — B cells from WT and IL-10−/− mice were isolated by MACS. Splenocytes were labeled with the cell proliferation dye eFluor 450 (10 μM) and activated with soluble anti-CD3e (1 μg/ml) and soluble anti-CD28 (1 μg/ml) antibody. B cells were activated with CpG or additionally treated with NE or Isopr. for 4 h, before cocultured with activated autologous splenocytes. Nonactivated and nontreated B cells were used as control group. CD4+ T cell proliferation was monitored by flow cytometry (n = 5). The PI has been calculated by using the number of events measured in each division peak. Ordinary 1-way ANOVA was used for comparisons. n.s., not significant; **p < 0.01; ***p < 0.001; ****p < 0.0001. For underlying data, see S1 Data. ANOVA, analysis of variance; Isopr., isoproterenol MACS, magnetic-activated cell sorting; NE, norepinephrine; PI, proliferation index. (PDF) [file pbio.3001513.s013.pdf]
