# Supplementary material for: Endogenously produced catecholamines improve the regulatory function of TLR9-activated B cells
Source: PLoS Biol. 2022 Jan 24;20(1):e3001513. doi: 10.1371/journal.pbio.3001513 (PMC8786184; doi:10.1371/journal.pbio.3001513)
Supplement: S11 Fig — Splenic B cells and B cell–depleted splenocytes from CIA mice were isolated by MACS. Splenocytes were labeled with the cell proliferation dye eFluor 450 (10 μM) and activated with soluble anti-CD3e (1 μg/ml) and soluble anti-CD28 (1 μg/ml) antibody. Autologous B cells were cultured in the presence of CII antigen or peptide (50 μg/ml and 100 μg/ml) for 24 h before cocultured with activated, B cell–depleted splenocytes for 72 h at 37°C and 5% CO2 at ratio of 1:2 (B cells:Splenocytes) in a 96 well plate. CD4+ T cell proliferation was monitored by flow cytometry (n = 6). One representative histogram is shown. Ordinary 1-way ANOVA was used for comparisons. n.s., not significant; *p < 0.5; **p < 0.01; ****p < 0.0001. For underlying data, see S1 Data. Ag, antigen; ANOVA, analysis of variance; CII, type II collagen; CIA, collagen-induced arthritis; MACS, magnetic-activated cell sorting; Pep, peptide. (PDF) [file pbio.3001513.s012.pdf]

S11 Figure

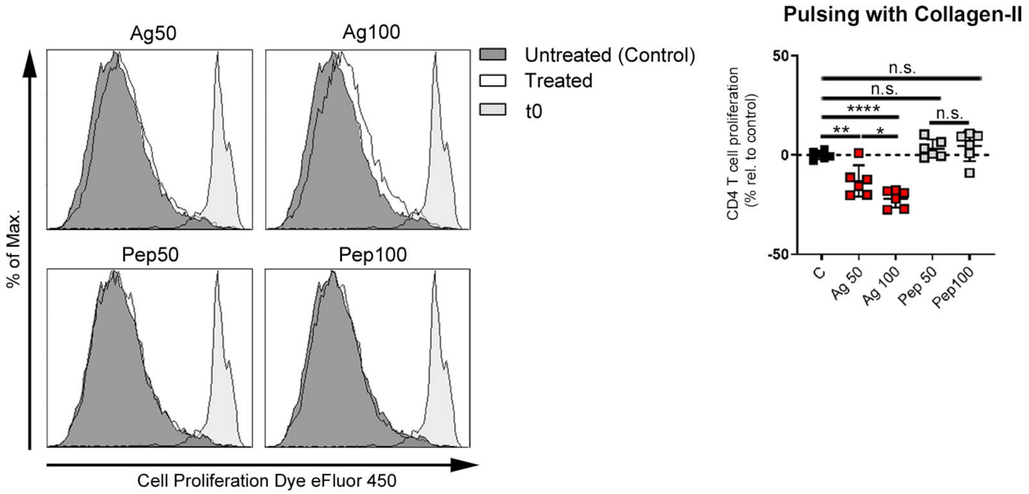

Ag50/100: Collagen-II (CII) antigen 50µg/ml or 100µg/ml

Pep50/100: Collagen-II (CII) peptide 50µg/ml or 100µg/ml
